# Feasibility of School-Based Identification of Children and Adolescents Experiencing, or At-risk of Developing, Mental Health Difficulties: a Systematic Review

**DOI:** 10.1007/s11121-020-01095-6

**Published:** 2020-02-15

**Authors:** Emma Soneson, Emma Howarth, Tamsin Ford, Ayla Humphrey, Peter B. Jones, Jo Thompson Coon, Morwenna Rogers, Joanna K. Anderson

**Affiliations:** 1 University of Cambridge Department of Psychiatry, Herchel Smith Building, Forvie Site, Robinson Way, Cambridge, CB2 0SZ UK; 2 NIHR Applied Research Collaboration (ARC) East of England, University of Cambridge, Cambridge, UK; 3 University of Exeter Medical School, Exeter, UK; 4 NIHR ARC South West Peninsula, University of Exeter Medical School, Exeter, UK

**Keywords:** Mental health, Schools, Identification, Screening, Feasibility

## Abstract

Under-identification of mental health difficulties (MHD) in children and young people contributes to the significant unmet need for mental health care. School-based programmes have the potential to improve identification rates. This systematic review aimed to determine the feasibility of various models of school-based identification of MHD. We conducted systematic searches in Medline, Embase, PsycINFO, ERIC, British Education Index, and ASSIA using terms for mental health combined with terms for school-based identification. We included studies that assessed feasibility of school-based identification of students in formal education aged 3–18 with MHD, symptomatology of MHD, or exposed to risks for MHD. Feasibility was defined in terms of (1) intervention fit, (2) cost and resource implications, (3) intervention complexity, flexibility, manualisation, and time concerns, and (4) adverse events. Thirty-three studies met inclusion criteria. The majority focused on behavioural and socioemotional problems or suicide risk, examined universal screening models, and used cross-sectional designs. In general, school-based programmes for identifying MHD aligned with schools’ priorities, but their appropriateness for students varied by condition. Time, resource, and cost concerns were the most common barriers to feasibility across models and conditions. The evidence base regarding feasibility is limited, and study heterogeneity prohibits definitive conclusions about the feasibility of different identification models. Education, health, and government agencies must determine how to allocate available resources to make the widespread adoption of school-based identification programmes more feasible. Furthermore, the definition and measurement of feasibility must be standardised to promote any future comparison between models and conditions.

**Electronic supplementary material:**

The online version of this article (10.1007/s11121-020-01095-6) contains supplementary material, which is available to authorized users.

## Background

Mental health difficulties (MHD) in children and young people (CYP) are an important public health challenge globally (Patel et al. 2007). MHD, including diagnosed psychiatric disorders, as well as subclinical symptoms of poor mental health (e.g. behavioural and socioemotional problems), are associated with a number of negative short- and long-term social, health, academic, and economic outcomes (Belfer 2008; Breslau et al. 2008; Green et al. 2005; Jokela et al. 2009). Whilst several early intervention strategies have shown success in reducing the burden of MHD in CYP (Children and Young People’s Mental Health and Wellbeing Taskforce 2015; Fazel et al. 2014; National Health Service England 2016), only about 15–30% of CYP with MHD receive any treatment (Burns et al. 1995; Eklund and Dowdy 2014; Kohn et al. 2004). Under-identification contributes to this gap: frontline gatekeepers such as teachers or primary care providers only identify 0.6–16% of CYP with MHD (Jensen et al. 2011; Levitt et al. 2007).

Improving rates of identification is important for increasing access to care and support for CYP with MHD. Schools are well placed to identify and support CYP with MHD due to their near universal access to CYP, high number of contact hours, close relationships with students and families, and the fact that the majority of MHD begin during the schooling years (Department of Health and Department for Education 2017; Humphrey and Wigelsworth 2016; Weist et al. 2007; Williams 2013). Furthermore, the recent UK Government Green Paper on CYP’s Mental Health sets expectations for schools to take a central role in the identification of and response to MHD (Department of Health and Department for Education 2017). Yet, despite these expectations, many school staff members feel unprepared to recognise MHD in their students (Day et al. 2017; Evans et al. 2016).

Schools currently use four main models to identify students with MHD (Panel 1). Universal screening refers to the assessment of all students using self-, teacher-, or parent-report measures. Selective screening is similar to universal screening but only assesses students with certain identifiable risk factors. Staff in-service training refers to increasing school staff’s knowledge and building capacity to recognise and refer students at-risk of or experiencing MHD. Curriculum-based models centre around educating students about MHD and rely on students to identify MHD and communicate concerns appropriately. In the UK, over 80% of schools currently rely on *ad hoc* identification of MHD. Systematic approaches are far less common; for example, only 15% of schools use universal screening, and a quarter use selective screening (NatCen Social Research and The National Children’s Bureau Research and Policy Team 2017).

School-based programmes have the potential to improve rates of MHD identification in CYP (Anderson et al. 2018). In the design and implementation of such programmes, it is important to consider not only effectiveness, but also social validity (Craig et al. 2008; Humphrey and Wigelsworth 2016). Social validity refers to ‘social importance’, or how much value society ascribes to the goals, procedures, and effects of a given programme (Wolf 1978). The social validity of an identification programme may refer to its feasibility, acceptability, and utility (Humphrey and Wigelsworth 2016), and is key for promoting successful implementation and long-term sustainability.

In this review, ‘feasibility’ refers to the impact of factors that affect programme implementation, including demand, ease of delivery, practicality, flexibility, and some aspects of acceptability (Bird et al. 2014; Bowen et al. 2009). Barriers and facilitators at the intervention level as well as the larger context in which an intervention takes place (e.g. school or policy context) can affect implementation and sustainability (Domitrovich et al. 2008; Ozer 2006). Understanding these barriers and facilitators is important for scaling up evidence-based mental health interventions in schools (Fazel et al. 2014). To be sustainable, programmes must be feasible for all stakeholders, including students, parents, school staff, and mental health professionals.

Despite the clear importance of, and recent policy focus on, early identification of MHD, there is a paucity of evidence for the effectiveness, feasibility, and acceptability of school-based identification models, especially within the UK (Fazel et al. 2014; Humphrey and Wigelsworth 2016). A recent systematic review (Anderson et al. 2018) examined the effectiveness of school-based models of identification. The present linked review sought to determine the feasibility of various models of school-based MHD identification.

## Methods

The protocol for this review is registered with the International Prospective Register of Systematic Reviews (PROSPERO; https://www.crd.york.ac.uk/prospero; registration number: 42016053084 (18 January 2017 version)).

### Definition of Feasibility

At the outset of this review, a literature search returned only one systematic tool for measuring the feasibility of mental health interventions: the Structured Assessment of FEasibility (SAFE) tool (Bird et al. 2014) (additional frameworks have been developed and tested since; see Weiner et al. (2017)). The SAFE tool features sixteen different aspects of feasibility, which we adapted (excluding ‘effectiveness’, ‘pilotable’, and ‘reversible’ criteria) to fit our research questions. To facilitate concise reporting, we further grouped the aspects of feasibility into four headings:

1. Intervention fit: matches prioritised goals, applicable to population of interest
2. Cost and resource implications: costly setup, cost-saving, additional human resources, additional material resources, staff training, on-going supervision
3. Intervention complexity, flexibility, manualisation, and time concerns
4. Adverse events

### Inclusion and Exclusion Criteria

We included studies that assessed feasibility of school-based interventions to identify students aged 3–18 years with (1) diagnosable MHD, (2) symptoms of mental ill health, or (3) exposure to psychosocial risk leading to increased risk of MHD. Most of the above feasibility categories are inherently informative; for example, whether a programme was perceived as too complex has clear, direct implications for feasibility. However, for three categories—staff training, on-going supervision, and manualisation—we required a more detailed comment than whether or not a programme included these elements (i.e. a statement about the implications of these elements on a programme’s implementation or sustainability). We excluded studies that focused on cost-effectiveness, as this outcome is included in our linked review (Anderson et al. 2018). We had no restriction regarding informants about feasibility. We included studies that did not explicitly state that they were measuring feasibility, but did report on at least one of the outcomes listed above, and studies that included in-principle findings (i.e. did not examine a specific intervention). We excluded studies that focused on global or specific learning disabilities or psychometric properties of an identification measure. We did not restrict study design.

### Search Strategy

We searched the following electronic databases in May and June 2017 and again in July 2018: Medline and Embase via OvidSP; PsycINFO, ERIC, and British Education Index via EBSCOhost; and ASSIA via ProQuest. The search strategy (Supplementary Table 1) included two domains: school-based identification and mental health. We combined our search terms with subject heading terms in each database. We collected additional citations through hand-searching reference lists of key publications and relevant journals.

### Study Selection and Data Extraction

Independent reviewers (ES, JKA, EH) double screened studies in three stages: (1) reviewers screened titles and removed obviously irrelevant citations; (2) reviewers judged abstracts against inclusion/exclusion criteria; and (3) reviewers examined full texts of potentially relevant citations against inclusion/exclusion criteria. We resolved disagreements by discussion. Two reviewers (ES, JKA) piloted data extraction tables with three studies to ensure they captured all relevant information. The reviewers independently extracted data; disagreements were solved by discussion, and if necessary by a third reviewer (EH). We extracted information on study design, study aims, school level(s), identification measures, informants, programme descriptions, and sample characteristics. Regarding feasibility, we extracted outcomes according to the SAFE categories, feasibility informants, and the method of determining feasibility.

### Critical Appraisal

We assessed quantitative studies using the Canadian Effective Public Health Practice Project (EPHPP) Quality Assessment Tool for Quantitative Studies (Armijo-Olivo et al. 2012) and qualitative studies using the Critical Appraisal Skills Programme (CASP) Qualitative Research Checklist (Critical Appraisal Skills Programme (CASP) 2013). We used both tools to assess mixed methods studies.

### Synthesis of Results

We provide a numerical account of included studies and employ narrative synthesis to present results, with studies grouped based on the type of identification model evaluated. We use Popay et al.’ (2006) guidance on narrative synthesis to guide reporting and provide a summary and conclusions in the discussion (Popay et al. 2006).

## Results

**Fig. 1:**
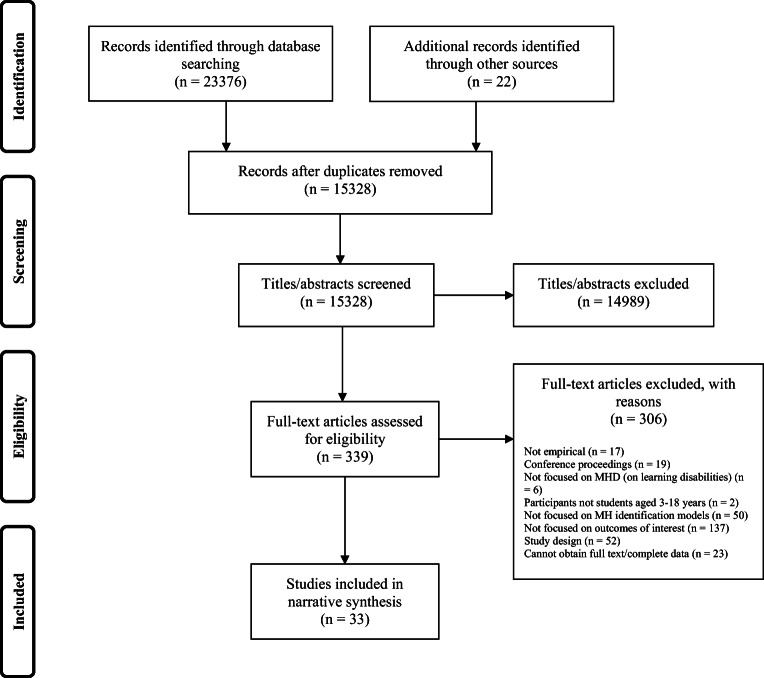
Study selection and exclusion flow diagram

Thirty-three studies met inclusion criteria (see Figure 1 for PRISMA flowchart and Supplementary Table 2 for an account of studies). The vast majority were conducted after the year 2000 (*n* = 29) and were from the United States(*n* = 27). Most studies used a cross-sectional design to assess feasibility (*n* = 26) and examined universal screening (*n* = 30). Behavioural and socioemotional problems (*n* = 14) and suicide risk (*n* = 11) were the most-studied conditions.

### Quality of Included Studies

#### Quantitative Studies

We provide quality ratings in Supplementary Table 3. The overall methodological quality of included studies was low. The majority were rated ‘weak’ in study design (*n* = 25) and had ‘moderate’ risk of selection bias (*n* = 21). Only seven studies used validated and reliable tools to measure feasibility. Drop-out rates varied between studies.

#### Qualitative Studies

Four of the studies with qualitative elements (D’Souza et al. 2005; Kirk 2014; Nadeem et al. 2016; Whitney et al. 2011) scored well on the CASP tool, indicating appropriate research design, recruitment, data collection, and data analysis. Each study also included a clear statement of aims and findings and added value to the evidence base. One study (Gilmore et al. 2004) did not score as highly, due to lack of a clear aim and insufficiently rigorous data analysis.

### Feasibility of School-Based Identification of Mental Health Difficulties

We present characteristics of included studies in Table 1. We present feasibility findings and feasibility reporting by study in Supplementary Tables 4 and 5, respectively.

**Table 1:** Characteristics of included studies

1st Author(s) (year); country Condition	Study design^1^	Study aims	School level(s) Informants	Identification measure(s)	Study description Follow-up mechanism for students identified as having MHD/risk for MHD	Sample characteristics	Percentage of students identified as having MHD/risk for MHD
Universal and selective screening							
Barry et al. (2016); USA ADHD	Cross-sectional	To explore the feasibility of school-based identification of children at-risk for ADHD and the communication of results and recommendations to parents	Elementary school Teachers	Vanderbilt AD/HD Diagnostic Teacher Rating Scale (VADTRS) School Intervention Questionnaire (SIQ)	(1) Teacher-report questionnaire (VADTRS); (2) teacher-report questionnaire (SIQ) for children identified as at-risk by the VADTRS Results fed back to parents with recommendation to see primary care provider for further evaluation	Students, *n* = 5772 (1st–5th grade; mean age 8.7 years; 66.4% male)	18.1%
Bruhn et al. (2014); USA Behavioural and socioemotional problems	Cross-sectional	To examine current screening practices and barriers to screening implementation in K-12 schools	Elementary school Middle school High school ND	NA	NA NA	School- or district-level administrators, *n* = 454 (53.3% male; 67.6% district administrators, 22.2% principals, 2.2% counsellors, 3.1% school psychologists, 2.4% vice principals, 2.4% other)	NA
Chartier et al. (2008); USA Behavioural and socioemotional problems	Interrupted time series	To examine the difference in participation rates in a school-wide screening programme (the Developmental Pathways Screening Program) under passive versus active parental consent conditions	Middle school Students	Mood and Feelings Questionnaire (MFQ)	(1) Student-report questionnaire (MFQ); (2) students who scored above cut-off received clinical evaluation Results fed back to parents with referrals made to school- and community-based services as appropriate	2002–2003: Students, *n* = 1011 (6th grade; no further characteristics provided) 2003–2004: Students, *n* = 1021 (6th grade; no further characteristics provided)	2002–2003, 13.7% 2003–4: 14.7%
Chatterji et al. (2004); USA Anxiety, depression, substance use disorders	Economic evaluation and pre-post design	To use cost-analysis methods in a real-world setting To estimate costs of a school-based mental health screening and treatment programme over 2 years of operation	Middle school Students	Diagnostic Interview for Children (DISC) Predictive Scale (DPS) Children’s Global Assessment Scale (CGAS)	(1) Paper and pencil DPS; (2) DISC by an interviewer for all students who indicated suicidal behaviour or significant mood, substance use, or anxiety problems. *NB: in year 2, students completed the Voice DISC if they spoke English (interviewer for non-English speaking students) and only new students (mostly sixth graders) were screened*. In case of suicidal behaviours on DPS, students were screened by screening director and upon confirmation were seen by a psychiatrist (evaluation using CGAS). In case of depression, anxiety, or substance use disorders on DPS, students completed depression portion of DISC (if marked anxiety on DPS, then completed anxiety portion of DISC; same for substance use) Results fed back to parents with referral to school-based mental health services for individual or group counselling (most common referral type) or addition to waiting list for school- or community-based services as appropriate	Year 1 Students, *n* = 1155 (grades 6, 7, 8) Year 2 Students, *n* = 453 (mostly grade 6)	ND (10.0% and 2.4% of screened students referred to treatment in years 1 and 2, respectively)
Curtis et al. (2014); USA Substance abuse	Cross-sectional	To assess the feasibility and economic sustainability of conducting screening, brief motivational counselling intervention and referral to treatment (SBIRT) in two urban schools	Middle school High school Students	CRAFFT Screening Tool For Adolescent Substance Abuse (CRAFFT)	(1) Student-report interactive screening instrument (CRAFFT) Students with ‘some risk’ received a brief motivational interview and could receive recommendation for continued sessions. Students with ‘significant risk’ had their results fed back to parents, were offered a brief intervention, and were referred to formal treatment as appropriate	Students, *n* = 248 (6th–12th grade; *n* = 106 middle school, *n* = 135 high school; 47.1% male)	42% (25% at ‘moderate’ risk; 18% at ‘significant’ risk)
Davis (2014); USA Behavioural and socioemotional problems	Cross-sectional	To compare teacher nomination process with the BASC-2 Behavioral and Emotional Screening System (BESS) for the detection of students with emotional and behavioural disorders	Middle school Teachers	Teacher Nomination Form (TNF) Behavior Assessment System for Children, Behavioral and Emotional Screening System (BASC-2 BESS)	(1) ‘First gate’ nomination/ranking of 10 students likely to have emotional and behavioural disorders (5 externalising, 5 internalising); (2) ‘second gate’ teacher-report questionnaire (BASC-2 BESS) for top 5 ranked externalising and top 5 ranked internalising students Results fed back to teachers	Students, *n* = 2323 (51.3% male; no further information provided) Teachers, *n* = 59 (23% male; mean years teaching = 9.45 (school 1), 12.14 (school 2))	ND (*NB:* author treated each nomination as a *separate student*, even if multiple teachers nominated the same student. 74% of total *nominations* screened positive.)
Donohue et al. (2015); USA Behavioural and socioemotional problems	Cross-sectional	To evaluate the process and outcomes of a school counsellor-led universal screening programme in one school district	Elementary school Middle school High school Students	Behavior Assessment System for Children, Behavioral and Emotional Screening System (BASC-2 BESS)	(1) Student-report questionnaire (BASC-2 BESS) Results fed back to parents (for at-risk students only) with information on available school and community support. Further assessment (if necessary) and group- or individual-level counselling provided for at-risk students. Regular meetings between counsellors, teachers, administrators, and special education professionals to discuss and monitor students	Students, *n* = 94 (grades 3, 4, 6, 7, 9, 10; no further characteristics are provided)	9–10% (across 2 years of screening)
D’Souza et al. (2005); USA Eating disorders	Mixed methods	To evaluate the implementation and effectiveness of the high school version of the National Eating Disorders Screening Program	High school Students	National Eating Disorders Screening Program (NESDP) screening form—includes Eating Attitudes Test (EAT-26)	(1) Student-report questionnaire (NEDSP questionnaire) Results fed back to students with recommendation to see a clinician about eating disorder symptoms as appropriate	Students, *n* = 1027 (9th–12th grade; mean age = 15.9 years; 42% male) School staff, *n* = 4 (no further characteristics provided)	30% of girls and 16% of boys met criteria for clinical evaluation
Edmunds et al. (2005); UK Behavioural and socioemotional problems	Cross-sectional	To examine the feasibility of the Child Health Assessment at School Entry (CHASE) questionnaire To assess the acceptability of the questionnaire to parents, teachers, nurses To examine quality of obtained data and quantify the validity and reliability and of the questionnaire	Primary school Parents, school nurses	CHASE questionnaire comprising Strengths and Difficulties questionnaire (SDQ) and Child Health Questionnaire - Parent Form 28 (CHQ-PF28) School nurse questionnaire using school health and education records	(1) Parent-report questionnaire (CHASE questionnaire) and school nurse questionnaire (in no defined order) ND	Students, *n* = 278 (year 1; no further characteristics provided) School nurses, *n* = 7 (no further characteristics provided)	SDQ ‘borderline’ score, 6.7% SDQ ‘abnormal’ score, 7.9%
Fox et al. (2013); USA Depression and suicide risk	Cross-sectional	To examine parental attitudes regarding school-based depression and suicide screening and education To identify predictors of positive perceptions of screening	Elementary school Middle school High school ND	NA	ND NA	Parents, 511 (50.9% male; mean age = 44.9 years)	NA
Gilmore et al. (2004); New Zealand Behavioural problems	Mixed methods	To develop and evaluate a screening and intervention model at school entry	Primary school Teachers, keyworkers	Brief Behaviour Screening Checklist (5-item) Behaviour Screening Checklist (28-item)	(1) Collaborative interview with teacher and keyworker (Proactive Screening Meeting; PSM); (2) teacher-report Brief Behaviour Screening Checklist for all children; (3) Behaviour Screening Checklist for children who are of concern during the PSM Individual, group, class, and school-wide in-school interventions (implemented by teachers, with parent involvement). Some ‘home interventions’	Students, *n* = 15 (no further characteristics provided)	ND
Gould et al. (2005); USA Suicide risk	RCT	To determine whether there is an iatrogenic effect of screening for suicide risk, i.e. does screening increase suicidal ideation or distress among (a) general population of high school students or (b) high-risk population of students	High school Students	Profile of Mood States (POMS-A) Suicidal Ideation Questionnaire (SIQ-JR) Interim Depression and Suicidal Ideation Beck Depression Inventory (BDI) and Drug Use Screening Inventory (DUSI) and Suicide Attempt History	2-day screening strategy: (day 1) all students completed POMS-A, BDI, DUSI, and second POMS-A; students in experimental group additionally complete SIQ-JR and suicide attempt history; (day 2) all students completed another POMS-A, an interim depression question, and 4 suicidal ideation measures (SIQ-JR, suicide attempt history, interim suicide item, BDI suicide item) Further interview for students reporting serious distress, serious suicidal ideation, and suicide attempt. Referrals to treatment arranged as needed with parent involvement	Students, *n* = 2342 (*n* = 1172 intervention and *n* = 1170 control) (9th–12th grade; mean age = 14.8 years)	ND (percentages of students screening positive for depression/suicidal ideation are given only for the interim period between days 1 and 2)
Hallfors et al. (2006a); USA Suicide risk	Cross-sectional	To assess the feasibility of a school- and population-based approach for suicide prevention in adolescents	High school Students	School records (i.e. a combination of absences and GPA) and teacher referral High School Questionnaire (HSQ) audio computer-assisted format Suicide Risk Screen (SRS)	(1) HSQ used to determine which students were high risk: (a) in upper 25% of distribution of absences AND lower 50% of grade point average, GPA, or (b) nominated by a teacher; (2) Student-report questionnaire (SRS) completed by typical and at-risk students Follow-up interview conducted by school staff. Referrals made as necessary, and parents were given lists of community-based services.	Students, *n* = 1323 (9th–11th grade; 48.1% male); *n* = 393 typical students; *n* = 930 high-risk students	29%
Hallfors et al. (2006b); USA Substance use and related problems	Case control	To examine the performance of a school-based screening method that uses school record data and teacher nomination	High school Students	School records (i.e. a combination of absences and GPA) and teacher referral High School Questionnaire (HSQ) audio computer-assisted format Suicide Risk Screen (SRS)	(1) School records used to determine which students were high risk: (a) in upper 25% of distribution of absences AND lower 50% of grade point average, GPA, or (b) nominated by a teacher; (2) student-report questionnaire (HSQ) to assess risk behaviours (SRS embedded) ND	Students, *n* = 1323 (9th–11th grade; 48.1% male); *n* = 393 typical students; *n* = 930 high-risk students	High-risk group vs. ‘typical’ group Cigarette use, 24% vs. 9% Alcohol use, 49% vs. 27% Marijuana use, 31% vs. 15% Other illegal drug use, 15% vs. 13% Suicide risk, 34% vs. 18%
Hallfors et al. (2000); USA Substance (alcohol, tobacco, and other drugs) use	Cross-sectional	To test whether computer-assisted self-interviews (CASI) could be applied in public school settings to improve accuracy of substance use data To examine implementation, acceptability, and advantages of CASI	Middle school High school Students	Santa Barbara schools: Santa Barbara Student Substance Use Survey (adapted from the California State Substance Use Survey) Vallejo schools: American Drug and Alcohol Survey; Prevention Planning Survey	(1) Student-report measure (either CASI or paper and pencil; measure varied by school district) ND	Santa Barbara students, *n* = 1555 (grades 7, 9, 11) Vallejo students, *n* = 1874 (grades 7, 9, 11)	ND
Kirk (2014); USA Behavioural and socioemotional problems	Mixed methods	To compare three methods of screening for emotional and behavioural difficulties To explore teacher perspectives on the screening and examine screening acceptability	Elementary school Teachers	Behavior Assessment System for Children, Second Edition (BASC-2) BASC-2 Behavioral and Emotional Screening System (BASC-2 BESS) Teacher referral data and office discipline referrals (ODRs)	(1) Teacher-report questionnaires (BASC-2 BESS) for all students; BASC-2 for 5 randomly selected students; ODR and teacher referral data collected Results fed back to parents and to teacher, with parents’ permission (for at-risk students only) ‘Follow-up support’ provided by principal and school counsellor, where needed	Students, *n* = 109 (kindergarten–6th grade; 53% male; no further characteristics provided) Teachers, *n* = 13 (8% male; mean years of teaching = 15.1, range 2–29)	Screening with BASC-2 BESS, 21% Teacher nomination, 25% ODRs method, 5%
Lyon et al. (2016); USA Depression	Modelling study	To provide an example of the utility of system dynamics modelling To explore how system dynamics modelling can be used to inform decisions in school-based depression screening by identifying (1) components that can influence delivery and outcomes, (2) additional resource requirements, and (3) leverage points providing opportunity for addressing mental health needs	High school Students	Moods and Feelings Questionnaire (MFQ)	(1) Student-report questionnaire (MFQ); (2) assessment and referral by mental health provider Model assumes mental health and non-mental health intervention options are available to identified students (model focuses on Interpersonal Therapy for Adolescent Depression as key mental health treatment)	Model assumes *n* = 1000 students	Model assumes 13.9% of students may score ‘high’ for depression
McManus (2009); USA Behavioural and socioemotional problems	Cross-sectional	To evaluate the implementation of a social-emotional screening programme and how training, coaching, and monitoring of implementation affected teacher behaviour and child outcomes	Elementary school Teachers, parents	Ages and Stages Questionnaires: Social Emotional (ASQ:SE)	(1) Questionnaire (ASQ:SE) completed by teachers; (2) Teachers assist parents in the completion of ASQ:SE in home visits Further assessment and individualised social-emotional/behavioural support for students identified as ‘at-risk’	Students, *n* = 141 (ages 3–5 years; 41% male) Parents, *n* = 141 (76% biological mothers, 7% biological fathers, 17% other relatives) Teachers, *n* = 8 (3.5–29 years experience in Head Start programme)	ND
Nemeroff et al. (2008); USA Behavioural and socioemotional problems	Cross-sectional	To evaluate the feasibility of on-going school-based identification models for mental health problems	Middle school Junior school High school Students	Voice Diagnostic Interview Schedule for Children IV (DISC-IV) Mental Health Tracking Form (MHTF)	(1) Counsellors had option to use Voice DISC-IV as part of student assessments (recording information in MHTF) Results fed back to parent. Students identified as at-risk received recommendations for clinical evaluation with partnered clinics	Students, *n* = 530 (aged 9–18 years; no further characteristics provided) School counsellors and mental health staff, *n* = 41 (mean years counselling experience = 12.5; 19% male)	72% of *students evaluated by counsellor* (*NB:* schools could choose to use programme as selective or universal screening)
Poulsen et al. (2015); Australia Behavioural and socioemotional problems (post-disaster)	Cross-sectional	To gauge parent satisfaction with post-disaster screening To determine if satisfaction was related to following through of screening recommendations To run subgroup analyses for these variables using exposure to disaster, parent concern, and demographic characteristics	Primary school Middle school Secondary school Parents	Post-disaster Screening Evaluation UCLA Posttraumatic Stress Reaction Index (UCLA PTSD-RI) Children’s Depression Inventory - Short version (CDI-S) Spence Children’s Anxiety Scale (SCAS)	(1) Parent-report questionnaires (Post-disaster Screening Evaluation, UCLA PTSD-RI, CDI-S, SCAS) Results fed back to parents with recommendations for further assessment/treatment as appropriate	Students, *n* = 224 (aged 7–18 years; mean age = 11.0 years; 55% male) Parents, *n* = 130 (13.1% male; no further characteristics provided)	Moderate distress, 18.3% Severe distress, 19.6%
Robinson et al. (2011); Australia Suicide risk	RCT	To implement an early identification programme for students at-risk for psychological distress, deliberate self-harm, or suicidal ideation. To determine whether there are associated iatrogenic effects. To assess the acceptability of the programme.	High school Students	General Health Questionnaire (GHQ) Profile of Mood States-A (POMS-A)	(1) Brief online student-report questionnaire completed over 2 days (students completed half on 1 day, half on the second). Half of the class completed the half with a screening question about distress/self-harm/suicidal ideation on the first day; half completed this half on the second day; (2) brief suicide/self-harm awareness workshop; (3) student-report questionnaires (GHQ, POMS-A) At-risk students received clinical interviews with a member of the research team, along with referral to support as appropriate	Students, *n* = 272 (year 10; aged 14–16 years; all male)	11.4%
Romer (2012); USA Risk for behavioural or socioemotional problems	Cross-sectional	To evaluate the validity of the Social-Emotional Assets and Resilience Scales - (Student Short Forms) for the identification of middle school students at-risk for social/behavioural or mental health difficulties	Middle school Students, teachers	The Social-Emotional Assets and Resilience Scales - Short Form (SEARS-SF) Youth Self-Report (YSR) Behavioral and Emotional Screening System (BESS) teacher form	(1) Phase I: student-report questionnaire (SEARS-SF); (2) Phase II: 106 students (45 at-risk and 61 not at-risk) completed YSR and SEARS-SF; teachers completed behaviour rating scales on participating students (BESS, SEARS-SF); student records used to collect ODRs, absences, and other information	Students, *n* = 1176 (characteristics reported on a school-by-school basis: 6–8th grade; ages 10–15; 43.6–51.5% male)	21.7%
Shortt et al. (2006); Australia Risk for mental health difficulties	Pre-post	To evaluate screening programme in terms of teachers’ ability to identify at-risk students and intervene To explore the acceptability and feasibility of the RAMP programme	Primary school Secondary school Teachers	RAMP screening form (no further description given)	(1) Systematic screening form (RAMP) Individualised action plans for at-risk students, which may contain in-school support, school-family-community linkage, and/or referral to specific external mental health services	Students, *n* = 422 primary school students (years 1–6); *n* = 61 secondary school students (years 7–10) no further characteristics provided. (*NB: n* = 483 students screened as part of the programme) School staff, *n* = 34 primary school staff; *n* = 18 secondary school staff. No further characteristics provided	Total screened positive ND (*n* = 52 *newly identified* students of 483 screened)
Vander Stoep et al. (2005); USA Behavioural and socioemotional problems	Cross-sectional	To evaluate the feasibility, acceptability, and yield of the Developmental Pathways Screening Program (DPSP)	Middle school Students	Developmental Pathways Screening Questionnaire (DPSQ), which contains items from Mood and Feelings Questionnaire (MFQ) and Youth Self Report (YSR)	(1) Student-report questionnaire (DPSQ); (2) school-based clinical assessment using DISC-IV for all students who scored positive for emotional distress Results fed back to parents with referral as appropriate to interventions including academic tutoring, in-school counselling, and external mental health services	Students, *n* = 861 (6th grade; 54.2% male)	15.2%
Walker et al. (1994); USA Behavioural and socioemotional problems	Cross-sectional	To validate the results of the Systematic Screening for Behavior Disorders (SSBD) in an additional, non-norming site	Elementary school Teachers	Systematic Screening for Behavior Disorders (SSBD) Social Skills Rating System (SSRS) Office discipline referrals	(1) Stage 1: teacher nomination whereby teachers listed and ranked top 10 students exhibiting externalising behaviours and top 10 exhibiting internalising behaviours. (2) Stage 2: teacher-report Critical Events Index and Combined Frequency Index for adaptive/maladaptive behaviours. (3) Stage 3: direct observation of behaviours Referral for further assessment as appropriate	Students, *n* = 1468 (58 of which were previously diagnosed with behavioural disorder and served as comparison group; no further characteristics are provided) Teachers and staff, *n* = 57 Special education resource teachers and psychologists, *n* = 8	Stage 1: 32.4% Stage 2: 15.3% (of the original sample)
Staff in-service training							
Nadeem et al. (2016); USA Suicide risk	Qualitative	To explore school personnel perspectives on parental involvement a district-wide suicide prevention programme	Middle school School personnel	ND	Youth Suicide Prevention Programme: (1) annual trainings for school-staff programme psychologist to develop skills to identify and refer at-risk students Students ‘in crisis’ receive immediate support; schools contact parents and provide referrals to specialist services. Post-intervention phase includes developing in-school supports for students, following up with parents/external services, and facilitating school re-entry	School staff, *n* = 45 (*n* = 7 mental health counsellors, *n* = 2 nurses, *n* = 26 teachers, *n* = 10 administrators; 42% male; mean years in education = 14)	ND
Sayal et al. (2006); UK ADHD	Cross-sectional	To examine the impact of an educational intervention for teachers to promote better recognition of ADHD	Primary school Teachers, parents	Strengths and Difficulties Questionnaire (SDQ) hyperactivity scale	(1) Teacher recognition of ADHD based on DSM-IV criteria; (2) SDQ screening (parent/teacher informants); (3) interactive teacher training including description of ADHD, presentation at school, ADHD as a risk factor, possible outcomes, importance/pervasiveness of symptoms, differential diagnoses/comorbidity, information about medication/classroom management strategies; (4) teacher recognition of ADHD ND	Teachers, *n* = 96 Students, *n* = 2672 (mean age = 7.87, range 4–11 years; 50% male)	Teacher recognition at baseline, 3.2% SDQ screening, 3–4% Teacher recognition after training, 4.1% (*NB:* estimates for ‘probable’ ADHD)
Curriculum-based model							
Kalafat and Elias (1994); USA Suicide risk	Cross-sectional	To assess the efficacy of a high school suicide curriculum	High school Students	NA	(1) Education sessions for faculty, staff, and parents; training on procedure for responding to identified risk; establishment of links to community agencies; (2) half of students receive suicide awareness training in first marking period; half receive physical education classes (without suicide curriculum); (3) schedules reversed in the second marking period Curriculum model included lesson plans for 3 40–50 min participatory lessons: 1st lesson: information on suicide, attitudes toward suicide, tunnel thinking 2nd lesson: warning signs, roleplay with help-seeking focus 3rd lesson: video of consequences of not responding to peers, overview of school resources ND	Students, *n* = 253 (grade 10; 57% male)	ND
Comparative—universal screening vs. staff in-service training vs. curriculum based							
Eckert et al. (2006); USA Suicide risk	Cross-sectional	To examine the acceptability to students of three school-based suicide prevention programmes	High school ND	NA	Curriculum based: (1) school psychologist to provide information on suicide (warning signs, incidence, etc.); (2) school psychologist to assess students identified as ‘at-risk’ Staff in-service training: (1) staff receive 2-h presentation on suicide prevention at beginning of school year; (2) school psychologist to assess students identified as ‘at-risk’ School-wide screening: (1) self-report rating scale; (2) school psychologist to assess students identified as ‘at-risk’ Results fed back to parents (for at-risk students only) with referral information	Students, *n* = 662 (freshmen in university; mean age = 17.99; 24.5% male)	NA
Eckert et al. (2003); USA Suicide risk	Cross-sectional	To explore school psychologists’ perceptions of three different models of school-based suicide prevention programmes	High school ND	NA	Curriculum based: (1) school psychologist to provide information on suicide (warning signs, incidence, etc.); (2) school psychologist to assess students identified as ‘at-risk’ Staff in-service training: (1) staff receive 2-h presentation on suicide prevention at beginning of school year; (2) school psychologist to assess students identified as ‘at-risk’ School-wide screening: (1) self-report rating scale; (2) school psychologist to assess students identified as ‘at-risk’ Results fed back to parents (for at-risk students only) with referral information	School psychologists, *n* = 211 (31.7% male)	NA
Miller et al. (1999); USA Suicide risk	Cross-sectional	To explore high school principals’ perceptions of three different models of school-based suicide prevention programmes	High school ND	NA	Curriculum based: (1) school psychologist to provide information on suicide (warning signs, incidence, etc.) in 2-h slot; (2) school psychologist to assess students identified as ‘at-risk’ Staff in-service training: (1) staff receive 2-h presentation on suicide prevention at beginning of school year; (2) school psychologist to assess students identified as ‘at-risk’ School-wide screening: (1) self-report rating scale; (2) school psychologist to assess students who scored above predetermined cut-off Results fed back to parents (for at-risk students only) with referral information	High school principals, *n* = 185 (82.8% male)	NA
Scherff et al. (2005); USA Suicide risk	Cross-sectional	To explore school superintendents’ perceptions of three different models of school-based suicide prevention programmes	High school ND	NA	Curriculum based: (1) school psychologist to provide information on suicide (warning signs, incidence, etc.) in 2-h slot; (2) school psychologist to assess students identified as ‘at-risk’ Staff in-service training: (1) staff receive 2-h presentation on suicide prevention at beginning of school year; (2) school psychologist to assess students identified as ‘at-risk’ School-wide screening: (1) self-report rating scale; (2) school psychologist to assess students who scored above predetermined cut-off Results fed back to parents (for at-risk students only) with referral information	School superintendents, *n* = 210 (79.4% male)	NA
Whitney et al. (2011); USA Suicide risk	Qualitative	To explore school principals’ perceptions of school-wide identification models by examining three different models To examine barriers of implementation	Elementary school Middle school High school ND	NA	Curriculum based: (1) school psychologist to provide information on suicide (warning signs, incidence, etc.) in ~ 2-h slot; (2) school psychologist/counsellor to assess students identified as ‘at-risk’ Staff in-service training: (1) all staff receive ~ 2-h training on suicide prevention at beginning of school year from school psychologist/counsellor; (2) school psychologist to assess students identified as ‘at-risk’ School-wide screening: (1) brief (~ 10 min) self-report rating scale; (2) school psychologist/counsellor to assess students who scored above predetermined cut-off Results fed back to parents (for at-risk students only) with referral information	Public school principals, *n* = 7 (5/7 males; 3 high school, 1 middle school, 2 elementary school, 1 K-2 primary school)	NA

*NA* not applicable, *ND* not described

^1^Study designs represent the designs used to measure feasibility

#### Universal and Selective Screening

Thirty studies reported on the feasibility of universal or selective screening (Supplementary Table 5). Fourteen reported on screening programmes for behavioural and socioemotional problems (Bruhn et al. 2014; Chartier et al. 2008; Davis 2014; Donohue et al. 2015; Edmunds et al. 2005; Gilmore et al. 2004; Kirk 2014; McManus 2009; Nemeroff et al. 2008; Poulsen et al. 2015; Romer 2012; Shortt et al. 2006; Vander Stoep et al. 2005; Walker et al. 1994), eight on suicide risk (Eckert et al. 2003; Fox et al. 2013; Gould et al. 2005; Hallfors et al. 2006a; Miller et al. 1999; Robinson et al. 2011; Scherff et al. 2005; Whitney et al. 2011), four on substance abuse (Chatterji et al. 2004; Curtis et al. 2014; Hallfors et al. 2006b; Hallfors et al. 2000), three on depression (Chatterji et al. 2004; Fox et al. 2013; Lyon et al. 2016), and one each on ADHD (Barry et al. 2016), anxiety (Chatterji et al. 2004), and eating disorders (D’Souza et al. 2005).

##### Intervention Fit

Twenty-two studies considered whether screening programmes were applicable to students and fit with prioritised goals. From the perspective of school staff, screening for behavioural and socioemotional problems (Davis 2014; Gilmore et al. 2004; Kirk 2014; McManus 2009; Romer 2012; Shortt et al. 2006; Walker et al. 1994) and eating disorders (D’Souza et al. 2005) matched school priorities in practice. However, when asked about in-principle feasibility, staff did not view identification of such problems as a school responsibility (Bruhn et al. 2014). Similarly, four in-principle studies comparing different identification models found that school staff were not persuaded screening for suicide risk was beneficial or acceptable (Eckert et al. 2003; Miller et al. 1999; Scherff et al. 2005; Whitney et al. 2011), and questioned whether screening was within schools’ remit (Whitney et al. 2011). In these studies, staff preferred in-service training and curriculum-based models over screening. In practice, views on screening for suicide risk were mixed, with some staff finding it an acceptable model and others feeling it was not beneficial (Hallfors et al. 2006a; Robinson et al. 2011). Support from teachers and superintendents increased student participation in screening, particularly for programmes that parents did not view as important (Barry et al. 2016). In general, parental support for screening was strong; nearly all parents (84–89%) supported screening for depression and suicide risk (although support differed by ethnicity and parental history of mental illness) (Fox et al. 2013) and over 99% of parents were satisfied with a post-disaster screening programme for behavioural and socioemotional problems (Poulsen et al. 2015). Similarly, students and mental health professionals found it important to screen for risk for behavioural and socioemotional problems (Romer 2012; Shortt et al. 2006).

In terms of the relevance for students, school staff and parents generally viewed screening programmes for behavioural and socioemotional problems favourably (Davis 2014; Kirk 2014; McManus 2009; Nemeroff et al. 2008). In contrast, staff raised concerns about the applicability of programmes for suicide risk (Hallfors et al. 2006a; Miller et al. 1999) and eating disorders (D’Souza et al. 2005), believing that students would not take these screenings seriously. Indeed, the programme for eating disorders was more effective for female students than for male students, and boys generally viewed the programme less favourably than did girls (D’Souza et al. 2005). Similarly, in a programme for suicide risk, those at highest risk were less likely to find the programme helpful (Robinson et al. 2011). Compared with behavioural and socioemotional problems, conditions that received less support with respect to screening were less prevalent in students. For less common conditions, selective screening for smaller, higher-risk groups had greater acceptance (D’Souza et al. 2005; Hallfors et al. 2006b).

##### Cost and Resource Implications

Thirteen studies considered cost and resource implications of screening. Regardless of condition screened for, schools were concerned about programme sustainability due to human resource requirements from the data collection stage to the provision of on-going support for identified students (Bruhn et al. 2014; D’Souza et al. 2005; Donohue et al. 2015; Hallfors et al. 2006a; Hallfors et al. 2000; Vander Stoep et al. 2005; Whitney et al. 2011). These concerns were reflected in a modelling study that found depression screening would require additional mental health professionals in order to accommodate newly identified students (Lyon et al. 2016). Whilst several programmes offered training for school staff, only two reported on training feasibility. One study reported teacher training required only one hour (Barry et al. 2016), although another found that many staff declined training, believing their professional training to be sufficient (Hallfors et al. 2006a). Similarly, several programmes offered supervision to school staff, but only two studies commented on supervision feasibility. These studies found that on-going supervision was crucial and that school staff doubted programme sustainability in the absence of on-going support from research staff (D’Souza et al. 2005; Hallfors et al. 2006a). In terms of additional material resources, schools’ most common concern was about purchasing screening tools or equipment for computerised testing (Bruhn et al. 2014; Donohue et al. 2015; Hallfors et al. 2000; Vander Stoep et al. 2005).

Five studies commented directly on costs of screening with mixed findings. One study reported general concerns about schools’ budgets (Bruhn et al. 2014), whilst four reported absolute costs. Two studies on behavioural and socioemotional screening found relatively low costs: data collection alone cost £4.60 per student (Edmunds et al. 2005) and a full programme (including follow-up support) cost US$9–15 per student (Vander Stoep et al. 2005). However, two other studies reported much higher costs of US$149–194 per student screened (Chatterji et al. 2004; Walker et al. 1994). These costs were not clearly related to the identity of those who delivered the screening programme (i.e. school staff (Edmunds et al. 2005; Walker et al. 1994), research staff (Vander Stoep et al. 2005), or a combination of in-school/external staff (Chatterji et al. 2004)).

##### Intervention Complexity, Flexibility, Manualisation, and Time Concerns

Thirteen studies commented on the complexity of screening programmes. Not all programmes were perceived as complex (Gilmore et al. 2004; Hallfors et al. 2006b; McManus 2009), but when they were, common factors of difficulty included obtaining consent, persuading teachers to release student time to complete assessments, collecting and analysing data, and integrating programmes into school culture. Schools viewed active parental consent requirements as a significant barrier (Barry et al. 2016; Chartier et al. 2008; Kirk 2014); obtaining consent was particularly difficult for students of lower socioeconomic status (Barry et al. 2016) and for students at high risk for MHD (Chartier et al. 2008). There was no clear consensus regarding the preferred method or mode of data collection. Whilst some questionnaires were easy to complete (McManus 2009), others used difficult wording (Donohue et al. 2015). For school staff, there was also no consensus regarding whether screening was more feasible in computerised or traditional format, but students tended to prefer computerised assessment (Hallfors et al. 2000; Nemeroff et al. 2008). Perceptions of programmes requiring school data varied; some were viewed as complex (Edmunds et al. 2005) but others as relatively easy (Hallfors et al. 2006b), as determined by availability and ease of use of school records. School staff also found it difficult to fully comply with screening protocols (Hallfors et al. 2006a) and to integrate screening into existing structures (D’Souza et al. 2005).

Eighteen studies commented on time concerns. Across conditions, school staff believed programmes were time-prohibitive, and had difficulty finding time to administer questionnaires, enter and analyse data, follow-up with identified students, and integrate programmes into school culture (Barry et al. 2016; D’Souza et al. 2005; Donohue et al. 2015; Eckert et al. 2003; Edmunds et al. 2005; Gilmore et al. 2004; Hallfors et al. 2006a; Hallfors et al. 2000; Kirk 2014; Miller et al. 1999; Nemeroff et al. 2008; Scherff et al. 2005). Programmes that identified large numbers of students were more likely to be viewed as overly time-intensive due to follow-up requirements (Hallfors et al. 2006a). Only four studies (Curtis et al. 2014; Davis 2014; Hallfors et al. 2000; Robinson et al. 2011) reported that staff, parents, and students viewed screening as time-efficient, with computerised assessment helping to reduce time requirements (Hallfors et al. 2000). Four of the five studies that quantified time resources found that completion of questionnaires required 15–50 minutes (Curtis et al. 2014; Edmunds et al. 2005; McManus 2009; Vander Stoep et al. 2005) and follow-up with identified students required 10–30 minutes per student (Curtis et al. 2014), though one programme reported time requirements of 6.43 hours per student (Walker et al. 1994). Whilst school staff generally believed that this was reasonable, large numbers of identified students overwhelmed schools (Hallfors et al. 2006a).

Four studies evaluated screening programme flexibility, all of which found that school staff valued the ability to tailor programmes to fit schools’ and students’ needs. Schools adapted programmes both in terms of format (Curtis et al. 2014; D’Souza et al. 2005) and target population (Hallfors et al. 2006a; Nemeroff et al. 2008), which increased perceived feasibility.

##### Adverse Events

Only two studies reported on potential harms of screening, both of which concerned programmes that screened for suicide risk. These studies compared distress and suicidal ideation of students who were or were not exposed to questions about suicide, and found no significant difference in either distress or suicidal ideation between the groups (Gould et al. 2005; Robinson et al. 2011), including for students at high risk for suicide (Gould et al. 2005).

#### Staff In-service Training

Eight studies reported on the feasibility of staff in-service training, six of which focused on identification of suicide risk (Eckert et al. 2003; Eckert et al. 2006; Kalafat and Elias 1994; Miller et al. 1999; Nadeem et al. 2016; Scherff et al. 2005; Whitney et al. 2011) and one of which on ADHD (Sayal et al. 2006). All but two studies (Nadeem et al. 2016; Sayal et al. 2006) examined in-principle feasibility and all examined the views of school staff.

##### Intervention Fit

All eight studies commented on intervention fit. In general, teachers perceived in-service training for identifying ADHD as appropriate, relevant, and useful (Sayal et al. 2006). There was no clear consensus on whether in-service training for identifying suicide risk matched school priorities. Although staff questioned whether mental health should be the responsibility of schools (Nadeem et al. 2016; Whitney et al. 2011), they generally viewed in-service training as beneficial for students (Eckert et al. 2003; Miller et al. 1999; Scherff et al. 2005). Furthermore, although school staff believed that in-service training was appropriate for a variety of students (Miller et al. 1999), some evidence suggested that female students may find staff in-service training more acceptable than do males (Eckert et al. 2006). Finally, school staff expressed concern about teacher and parent buy-in (Nadeem et al. 2016; Whitney et al. 2011).

##### Cost and Resource Implications

Two studies commented on cost and resource implications of staff in-service training. School staff thought that resources for mental health were crucial, particularly for students without access to care outside of school (Nadeem et al. 2016). Staff thought that the training was valuable and that it would help them to identify and support students with mental health needs (Nadeem et al. 2016; Whitney et al. 2011).

##### Intervention Complexity, Flexibility, Manualisation, and Time Concerns

Two studies reported on complexity of staff in-service training programmes. School staff viewed in-service training as complex due to difficulties communicating with parents about their child’s risk (Nadeem et al. 2016). However, staff also believed that in-service training was easier to implement in comparison with other models of identification (i.e. curriculum-based models and universal screening) (Whitney et al. 2011). Three studies commented on time concerns. School staff viewed in-service training as intrusive into staff and student time (Eckert et al. 2003; Miller et al. 1999), although this was less of a concern for school superintendents (Scherff et al. 2005).

##### Adverse Events

Not described.

#### Curriculum-Based Models

Seven studies reported on the feasibility of curriculum-based models for identification of suicide risk (Eckert et al. 2003, 2006; Kalafat and Elias 1994; Miller et al. 1999; Scherff et al. 2005; Whitney et al. 2011), six of which examined in-principle feasibility according to school staff or students and one of which (Kalafat and Elias 1994) assessed in-practice feasibility according to students.

##### Intervention Fit

All seven studies commented on intervention fit. School staff generally agreed that curriculum-based models were beneficial, helpful, and appropriate for a variety of students (Eckert et al. 2003; Miller et al. 1999; Scherff et al. 2005). However, some doubted the fit for younger students and raised concerns about lack of teacher buy-in and parental objections (Whitney et al. 2011). In-principle perceptions varied among students, with female students finding curriculum-based models more acceptable and less intrusive than male students (Eckert et al. 2006). In practice, however, most students found the curriculum-based approach to identifying suicide risk to be useful, interesting, relevant, and important (Kalafat and Elias 1994).

##### Cost and Resource Implications

Not described.

##### Intervention Complexity, Flexibility, Manualisation, and Time Concerns

One study commented on programme complexity and found that school principals appreciated that curriculum-based models were easy to implement, standardised, and deliverable to all students (Whitney et al. 2011). Six studies reported on time concerns. In general, school staff were concerned about curriculum-based models intruding into staff and student time (Eckert et al. 2003; Scherff et al. 2005; Whitney et al. 2011), although school superintendents were less concerned about time requirements (Scherff et al. 2005).

##### Adverse Events

The only study to report on adverse events found that only 3% of students rated classes about suicide as upsetting (Kalafat and Elias 1994).

## Discussion

We identified 33 studies that reported on the feasibility of school-based identification of MHD. Most studies focused on behavioural and socioemotional problems or suicide risk were cross-sectional in design and examined feasibility from the perspective of school staff.

Screening programmes were the most common identification model evaluated. Most school staff perceived screening to be aligned with school priorities but viewed programmes that screened for less prevalent conditions (e.g. eating disorders, substance abuse, and suicide risk) as less applicable to all students. Across conditions, school staff were concerned about additional human and material resources, and costs varied widely (from less than £5 per student for data collection to nearly US$200 per student screened). Time concerns were common across models and conditions, and staff doubted whether schools had enough time to complete screening, particularly when the process involved following up with at-risk students. Attainment of consent and communication with parents were significant barriers to feasibility. Flexible programmes were reported as more feasible, particularly when universal screening could be adapted to target higher-risk groups only. No study found evidence of harms resulting from screening.

Staff in-service training and curriculum-based models were less common and most focused on suicide risk. In-service training matched well with school priorities and was helpful in principle, but in practice, many school staff doubted whether mental health was their responsibility, which might explain concerns about time and resource requirements. Curriculum-based models also aligned with school priorities and were perceived as helpful, standardised, and easy to implement. School staff generally viewed both models as intrusive into staff and student time.

Suicide risk and ADHD were the only conditions represented across two or more identification models, thereby providing opportunity for comparison. For suicide risk, school staff preferred in-service training and curriculum-based programmes to universal screening. Compared with screening, these models aligned more with prioritised goals and were perceived as more applicable to a variety of students and easier to implement. Screening was ubiquitously viewed as the most time-intrusive model. ADHD identification had similar trends, whereby school staff and parents viewed staff in-service training as a better fit than screening.

There were important differences between findings from in-principle studies and studies of specific interventions, with the former generally showing lower feasibility. In-principle studies found that MHD identification was less of a priority and that programmes were less applicable to students. This might be explained by the fact that studies of specific interventions would have taken place in schools for which identification was enough of a priority to participate in research and were therefore viewed more favourably. Alternatively, initial concerns may be allayed when a programme is delivered in practice.

### Quality of the Evidence

Although the majority of quantitative studies were rated ‘weak’ in terms of study design, Bowen et al. (2009) have argued that several designs besides RCTs are appropriate for assessing feasibility, including cross-sectional and pre-post designs (Bowen et al. 2009). Studies used a variety of methods to measure feasibility, including authors’ observations, surveys, rating tools, and interviews. However, few utilised validated and reliable measures to measure feasibility, which is unsurprising given the scarcity of available tools. The qualitative studies and qualitative elements of mixed methods studies were generally of high quality and examined feasibility in more depth by exploring context as well as the logic and reasoning underlying stakeholder perspectives.

### Limitations

We acknowledge several limitations. First, we only included studies published in English. Second, the lack of standardised definition and validated measures of feasibility limited our ability to compare feasibility across studies and identification models. Finally, whilst the SAFE guidance was comprehensive, it would have been useful to compare it with other tools (nearly 40 identified aspects of feasibility were left out of the tool; see Appendix DS1 of Bird et al. 2014). Widening the scope of feasibility criteria would likely have led to inclusion of additional studies.

### Implications for Practice

Although the evidence did not indicate one identification model as more feasible than others, we did identify a number of key barriers. Collaboration between schools and mental health professionals, as recommended by the Green Paper on CYP’s Mental Health (Department of Health and Department for Education 2017), may help address some of these concerns. For example, mental health professionals could consult with schools to reduce barriers such as complexity and training/supervision requirements and could further assist in following up with identified students. Sharing the responsibility of identification between health and education sectors would also address schools’ concerns that mental health is not their responsibility. Indeed, several other settings may complement schools in the identification of MHD, including primary care practices.

Cost and resource concerns are perhaps more difficult to address, as schools have limited budgets and resources. However, these concerns can be partially addressed through efficient use of existing resources. For example, using routinely collected school data could help identify specific groups of students at increased risk of MHD (Kuo et al. 2013), thereby increasing the positive predictive value of any screening tool. Furthermore, despite some evidence that programmes can be costly for both schools and society, it is clear that affordable programmes do exist; at an estimated US$9–15 per student for identification and one-to-one follow-up (Vander Stoep et al. 2005), the costs of school-based identification can be much lower than for specialist care (Snell et al. 2013). Such programmes also offer opportunity for early diagnosis and treatment, which can reduce the long-term costs of MHD (Williams 2013). Given the clear benefits of early identification, education, health, and government sectors must collaborate to most effectively allocate existing resources.

Finally, in creating feasible programmes, stakeholder participation is crucial. Encouragingly, in this review, the majority of included studies assessed feasibility by directly surveying or interviewing school staff, parents, and/or students. Involving stakeholders in all phases of intervention design, evaluation, and implementation yields better quality research and improved outcomes, and promotes better integration and sustainability, increased ownership, and greater cultural sensitivity (Brett et al. 2014; Wallerstein and Duran 2010).

### Directions for Future Research

The first steps toward a better understanding of the feasibility of school-based MHD identification are to (1) create a standardised definition of feasibility and its components and (2) clarify how to reliably measure intervention feasibility. The development of standardised measures (Weiner et al. 2017) is crucial for both assessing feasibility of individual programmes and comparing feasibility across programmes. Furthermore, as feasibility can differ in principle and in practice, it is important to examine the relationship between the two through continued evaluation in all stages of intervention research (e.g. with detailed process evaluations (Craig et al. 2008; Moore et al. 2015)) and to assess feasibility in conjunction with effectiveness.

Furthermore, because programmes that are feasible and effective in one school may not be in others, researchers should explicitly examine school and policy contexts and their interaction with the intervention itself (Domitrovich et al. 2008; Ozer 2006). Such research is likely best conducted through mixed methods approaches and requires a detailed understanding of both intervention components and broader structural factors (Howarth et al. 2016). An examination of service context is particularly needed; most included studies were US-based, limiting generalisability to countries such as the UK, where long wait times often prohibit timely access to services (Frith 2017).

Finally, future research should continue to explicitly examine the potential for harms and unintended consequences related to MHD identification, as many school staff are concerned about the possibility of iatrogenic effects (Evans et al. 2016). Potential harms must be weighed against the benefits of the programme in order to inform practice (Public Health England 2015).

## Conclusions

This is the first known systematic review of the feasibility of school-based MHD identification. The evidence base regarding feasibility is not robust enough to support programme scale-up, and between-study variation in definition and measurement of feasibility prohibits definitive conclusions about the most feasible identification model. Time, resource, and cost concerns are the most common barriers to feasibility. Education, health, and government agencies must work together to determine how to best allocate available resources to make the widespread adoption of identification programmes more feasible. Further research is needed regarding other possible contexts for identification, such as primary care or online screening.

## Electronic supplementary material

ESM 1 (DOCX 38 kb)

ESM 2 (DOCX 17.3 kb)

ESM 3 (DOCX 23.4 kb)

ESM 4 (DOCX 30.5 kb)

ESM 5 (DOCX 20.7 kb)

ESM 6 (DOCX 21 kb)

ESM 7 (DOCX 162 bytes)
