# Supplementary material for: Feasibility of School-Based Identification of Children and Adolescents Experiencing, or At-risk of Developing, Mental Health Difficulties: a Systematic Review
Source: Prev Sci. 2020 Feb 15;21(5):581–603. doi: 10.1007/s11121-020-01095-6 (PMC7305254; doi:10.1007/s11121-020-01095-6)
Supplement: Supplementary file 1 — (DOCX 38 kb) [file 11121_2020_1095_MOESM1_ESM.docx]

**Supplementary Table 1. MEDLINE search strategy**

| # | **Query** | **Limiters/Expanders** | **Last Run Via** | **Results** |
| --- | --- | --- | --- | --- |
| S7 | S5 AND S6 | Search modes - Boolean/Phrase | Interface - EBSCOhost Research Databases  Search Screen - Advanced Search  Database - MEDLINE | 3,561 |
| S6 | AB ( (screen* OR identif* OR detect* OR assessment OR nominat* OR “case finding” OR case-finding) N3 (teacher* or school* or kindergarten* or nursery* or academy* or pupil* or student* or pre-school* or preschool*) ) OR TI ( (screen* OR identif* OR detect* OR assessment OR nominat* OR “case finding” OR case-finding)) N3 (teacher* or school* or kindergarten* or nursery* or academy* or pupil* or student* or pre-school* or preschool* or reception*) ) | Search modes - Boolean/Phrase | Interface - EBSCOhost Research Databases  Search Screen - Advanced Search  Database - MEDLINE | 16,150 |
| S5 | S1 OR S2 OR S3 OR S4 | Search modes - Boolean/Phrase | Interface - EBSCOhost Research Databases  Search Screen - Advanced Search  Database - MEDLINE | 6,534,873 |
| S4 | TI ( ((“emotional health”) OR ((mental OR emotional OR psychosocial) N2 (wellbeing OR well-being or problem*)) OR psychopathology OR (mental health N2 ((problem* OR disorder* OR risk* or poor)) OR ((mental* OR psychiatric) N2 (ill* OR disorder*)) OR ((behaviour* OR behavior*) N2 (problem* OR disorder* OR risk*)) OR depress* OR anxiety OR suicid* OR stress* OR distress* OR drug* OR substance* OR (“eating disorder*”) or “ADHD” or “attention deficit” ) OR AB ( ((“emotional health”) OR ((mental OR emotional OR psychosocial) N2 (wellbeing OR well-being or problem*)) OR psychopathology OR (mental health N2 ((problem* OR disorder* OR risk* or poor)) OR ((mental* OR psychiatric) N2 (ill* OR disorder*)) OR ((behaviour* OR behavior*) N2 (problem* OR disorder* OR risk*)) OR depress* OR anxiety OR suicid* OR stress* OR distress* OR drug* OR substance* OR (“eating disorder*”) or “ADHD” or “attention deficit”) | Search modes - Boolean/Phrase | Interface - EBSCOhost Research Databases  Search Screen - Advanced Search  Database - MEDLINE | 6,530,832 |
| S3 | (MH "Attention Deficit and Disruptive Behavior Disorders") | Search modes - Boolean/Phrase | Interface - EBSCOhost Research Databases  Search Screen - Advanced Search  Database - MEDLINE | 2,361 |
| S2 | (MH "Psychopathology") OR (MH "Psychology, Educational") | Search modes - Boolean/Phrase | Interface - EBSCOhost Research Databases  Search Screen - Advanced Search  Database - MEDLINE | 7,647 |
| S1 | (MH "Mental Disorders") OR (MH "Anxiety Disorders") OR (MH "Disruptive, Impulse Control, and Conduct Disorders") OR (MH "Feeding and Eating Disorders") OR (MH "Mood Disorders") OR (MH "Substance-Related Disorders") OR (MH "Depressive Disorder") | Search modes - Boolean/Phrase | Interface - EBSCOhost Research Databases  Search Screen - Advanced Search  Database - MEDLINE | 315,585 |
