# Supplementary material for: Feasibility of School-Based Identification of Children and Adolescents Experiencing, or At-risk of Developing, Mental Health Difficulties: a Systematic Review
Source: Prev Sci. 2020 Feb 15;21(5):581–603. doi: 10.1007/s11121-020-01095-6 (PMC7305254; doi:10.1007/s11121-020-01095-6)
Supplement: Supplementary file 2 — (DOCX 17.3 kb) [file 11121_2020_1095_MOESM2_ESM.docx]

**Table 1.** Account of included studies

| Criterion | Characteristic | Number of studies (n=33) |
| --- | --- | --- |
| Year | 1991-2000 | 4 |
|  | 2001-2010 | 16 |
|  | After 2010 | 13 |
| Country | USA | 27 |
|  | Australia | 3 |
|  | UK | 2 |
|  | New Zealand | 1 |
| Study design used to measure feasibility | RCT | 2 |
|  | Case control | 1 |
|  | Mixed methods^1^ | 3 |
|  | Interrupted time series | 1 |
|  | Cross sectional | 21 |
|  | Pre-post | 1 |
|  | Economic evaluation | 1 |
|  | Modelling study | 1 |
|  | Qualitative | 2 |
| Identification model^2^ | Universal screening | 30 |
|  | Selective screening | 3 |
|  | Curriculum-based | 6 |
|  | In-service training | 8 |
| Informants^3^ | Students | 16 |
|  | Parents | 4 |
|  | Teacher/other school staff | 12 |
|  | Not specified | 6 |
| Condition^4^ | ADHD | 2 |
|  | Anxiety | 1 |
|  | Behavioural and socioemotional problems | 14 |
|  | Depression | 3 |
|  | Eating disorders | 1 |
|  | Suicide risk | 11 |
|  | Substance abuse | 4 |
| School level^5^ | Primary/elementary school | 13 |
|  | Middle school | 13 |
|  | Secondary/high school | 20 |
| Parent consent^6^ | Yes | 20 |
|  | No | 1 |
|  | ND | 6 |
| Student assent^6^ | Yes | 7 |
|  | No | 0 |
|  | ND | 20 |
| Follow-up clinical evaluation^6^ | Yes | 7 |
|  | No | 2 |
|  | ND | 18 |
| Informing students/parents/school about results^6^ | Yes | 15 |
|  | No | 0 |
|  | ND | 12 |
| Referral/recommendations following identification^6^ | Yes | 17 |
|  | No | 0 |
|  | ND | 10 |

ND – not described

^1^Both mixed methods studies were cross-sectional in nature. ^2^Some studies describe multiple models of identification. ^3^Some studies examined views from multiple informants. ^4^Some programmes identified multiple conditions. ^5^Some studies were conducted in multiple schools, at different school levels. ^6^Components are reported only for studies that examined specific identification programmes (vs. theoretical feasibility).
