# Supplementary material for: Feasibility of School-Based Identification of Children and Adolescents Experiencing, or At-risk of Developing, Mental Health Difficulties: a Systematic Review
Source: Prev Sci. 2020 Feb 15;21(5):581–603. doi: 10.1007/s11121-020-01095-6 (PMC7305254; doi:10.1007/s11121-020-01095-6)
Supplement: Supplementary file 3 — (DOCX 23.4 kb) [file 11121_2020_1095_MOESM3_ESM.docx]

**Supplementary Table 2. Quality ratings for quantitative and mixed methods studies**

| 1^st^ author (year); study design^1^ | Selection bias | Study design^1^ | Confounders | Blinding | Data collection^2,3^ | Drop out |
| --- | --- | --- | --- | --- | --- | --- |
| Barry (2016); cross-sectional | Weak | Weak | NA | NA | NA | Strong |
| Bruhn (2014); cross-sectional | Weak | Weak | NA | NA | Weak | Weak |
| Chartier (2008); interrupted time series | Moderate | Moderate | NA | NA | NA | Moderate |
| Chatterji (2004); economic analysis of pre-post study | Moderate | Moderate | NA | NA | Strong | Weak |
| Curtis (2014); cross-sectional | Moderate | Weak | NA | NA | NA | Strong |
| Davis (2014); cross-sectional | Weak | Weak | NA | NA | Weak | Weak |
| Donohue (2015); cross-sectional | Moderate | Weak | NA | NA | NA | Weak |
| D’Souza (2005); mixed methods | Moderate | Weak | NA | NA | Weak (survey)/NA (author observations) | Moderate |
| Eckert (2006); cross-sectional | Weak | Weak | NA | NA | Strong | Weak |
| Eckert (2003); cross-sectional | Moderate | Weak | NA | NA | Strong | Weak |
| Edmunds (2005); cross-sectional | Moderate | Weak | NA | NA | Weak | Moderate |
| Fox (2013); cross-sectional | Weak | Weak | NA | NA | Moderate | Weak |
| Gilmore (2004); mixed methods | Weak | Weak | NA | NA | Weak | Weak |
| Gould (2005); RCT | Moderate | Strong | Strong | Moderate | NA | Strong |
| Hallfors, Brodish (2006); cross-sectional | Moderate | Weak | NA | NA | NA | Weak |
| Hallfors, Cho (2006); case control | Moderate | Moderate | NA | NA | NA | Weak |
| Hallfors (2000); cross-sectional | Weak | Weak | NA | NA | NA | Weak |
| Kalafat (1994); cross-sectional | Moderate | Weak | NA | NA | Weak | Weak |
| Kirk (2014); mixed methods | Weak | Weak | NA | NA | Weak | Moderate |
| Lyon (2016); modelling | NA | Weak | NA | NA | Strong | NA |
| McManus (2009); cross-sectional | Moderate | Weak | NA | NA | Weak | Strong |
| Miller (1999); cross-sectional | Moderate | Weak | NA | NA | Strong | Weak |
| Nemeroff (2008); cross-sectional | Moderate | Weak | NA | NA | NA | Strong |
| Poulsen (2015); cross-sectional | Weak | Weak | NA | NA | Moderate | Weak |
| Robinson (2011); RCT | Moderate | Strong | Weak | Moderate | Strong | Strong |
| Romer (2012); cross-sectional | Moderate | Weak | NA | NA | Weak | Strong |
| Sayal (2006); cross-sectional | Moderate | Weak | NA | NA | Weak | Moderate |
| Scherff (2005); cross-sectional | Moderate | Weak | NA | NA | Strong | Weak |
| Shortt (2006); pre-post | Moderate | Moderate | NA | NA | Weak | Strong |
| Vander Stoep (2005); cross-sectional | Moderate | Weak | NA | NA | NA | Strong |
| Walker (1994); cross-sectional | Moderate | Weak | NA | NA | NA | Moderate |

^1^ Study designs (and associated ratings) reflect the designs used to measure feasibility (not the overall study design).

^2^ Data collection ratings refer to collection of information relating to feasibility (not identification of MHD).

^3^ *NA* signifies that feasibility was assessed by authors, and not through a specific measurement tool.
