# Supplementary material for: Feasibility of School-Based Identification of Children and Adolescents Experiencing, or At-risk of Developing, Mental Health Difficulties: a Systematic Review
Source: Prev Sci. 2020 Feb 15;21(5):581–603. doi: 10.1007/s11121-020-01095-6 (PMC7305254; doi:10.1007/s11121-020-01095-6)
Supplement: Supplementary file 4 — (DOCX 30.5 kb) [file 11121_2020_1095_MOESM4_ESM.docx]

**Supplementary Table 3. Feasibility findings (categorised by SAFE framework category numbers)**

| **1^st^ Author (Year)  Condition** | **Methods  Respondents** | **Findings** |
| --- | --- | --- |
| **UNIVERSAL & SELECTIVE SCREENING** | | |
| Barry (2016)  ADHD | Authors’ observations  NA | **Intervention fit**  9. Parent and teacher involvement limited by socioeconomic disadvantage: schools with higher proportion of low socioeconomic status students had lower involvement 14. Many parents refused referral (reasons included not believing the child had a problem); school with superintendent whose goals aligned with the programme's had the best district involvement (e.g. higher rates of consent)  **Cost & resource implications**  1. Staff training through role-play: 5-6 hours for project staff and 1 hour for teachers  **Complexity, flexibility, and time concerns**  2. Active consent as a barrier; parent contact information was not up to date; poor parent-teacher communication  3. Teachers completed only 70% of screeners for children who had consent, citing measure length and inconvenience |
| Bruhn (2014)  Behavioural and socioemotional problems | Survey  K-12 school- or district-level administrators | **Intervention fit**  14. Schools already do too many screenings and do not want another one; small schools felt as though screening would not be helpful for them; screening is not the responsibility of the school  **Cost & resource implications**  5. Screening would require hiring additional personnel 5, 6. Schools do not have support systems for identified children 6. Schools do not have access to screening tools 7. There is not enough money in the school budget for school-wide screening  **Complexity, flexibility, manualisation, and time concerns**  2. Schools unsure of how to use or interpret data from screening |
| Chartier (2008)  Behavioural and socioemotional problems | Authors’ observations  NA | **Complexity, flexibility, manualisation, and time concerns**  2: Active parent consent as a barrier for participation: with passive consent, 85% of children participated in screening but with active consent, only 66% participated (with significant decreases in those at increased risk for depression) |
| Chatterji (2004)  Anxiety, depression, substance use disorders | Economic cost analysis  NA | **Cost & resource implications**  5, 6, 7, 13. Costs of screening 'start up' year (programme development) were US$37,347 for the school and US$41,354 for society  5, 6, 7, 13. Costs of screening in Year 1 were US$168,053 (US$146 per student screened; US$1449 per student referred to treatment through screening) for the school and US$172,018 (US$149 per student screened; US$1483 per student referred to treatment through screening) for society  5, 6, 7, 13. Costs of screening in Year 2 (screening for grade 6/new incoming students only) were US$87,726 (US$194 per student screened; US$7975 per student referred to treatment through screening) for the school and US$106,125 (US$234 per student screened; US$9648 per student referred to treatment through screening) for society  *NB: costs are given in 2000 US dollars* |
| Curtis (2014)  Substance abuse | Authors’ observations  NA | **Complexity, flexibility, manualisation, and time concerns eComplexity, flexibility, manualisation, and time concerns**  3. Screening was completed in a single class period, with 15-20 minutes for students to answer screening questions and 10-30 minutes to discuss answers with counsellor  11. Programme could be tailored for individual students based on age, gender, ethnicity, language, read-aloud options |
| Davis (2014)  Behavioural and socioemotional problems | Screening System Social Validity Form (SSSVF): researcher-designed survey to assess social validity with 7-point Likert scale where 1 = strongly disagree and 7 = strongly agree (and free-responses)  Teachers | Screening for students at-risk for SEBC through the Teacher Nomination Form was feasible at their school: mean=5.32, mode=5  **Intervention fit**  9. This system was appropriate for middle school students: mean=5.53, mode=5 14. This screening system met the needs of screening for students at-risk for SEBC: mean= 4.93, mode=5  14. Teachers believed programmes must do more than identify; they must also respond to mental health difficulties  **Complexity, flexibility, manualisation, and time concerns**  3. This system was conducted in a timely manner: mean=5.81, mode=6 |
| Donohue (2015)  Behavioural and socioemotional problems | Authors’ observations  NA | **Intervention fit**  2, 9. Students struggled with the wording of the screening tool  **Cost & resource implications**  5. Required additional staff to support to facilitate interventions post-identification 6. Schools would like to streamline screening through use of electronic assessments, requiring new software  **Complexity, flexibility, manualisation, and time concerns**  2, 9. Students struggled with the wording of the screening tool  3. Data entry and analysis were time consuming |
| D'Souza (2005)  Eating disorders | Semi-structured interviews  School staff | **Intervention fit**  9. The programme was more effective for girls than for boys (in terms of helping them learn about eating disorders, change thinking about eating disorders/body image, and talk to friend about eating disorders)  9. 61% of females and 48% of males would recommend the programme to friends; 78% of females and 66% of males would recommend to other schools  9. Believed it would be better to selectively screen (e.g. by targeting only girls); teachers felt that boys believed the screening didn’t apply to them 14. Staff in all schools believed it was important to offer the screening programme  **Cost & resource implications**  4. Doubts about sustainability without on-going supervision and support 5. Difficult to recruit staff members to help in planning process  **Complexity, flexibility, manualisation, and time concerns**  2. Difficulty concerning integration of NEDSP curriculum into school curricula 3. Hard to find time to administer screening  11. Schools adapted screening programme to different formats, e.g. drama production; some staff felt it was hard to integrate eating disorder materials into the curriculum |
| Edmunds (2005)  Behavioural and socioemotional problems | Semi-structured feedback questionnaire  School nurses | **Cost & resource implications**  5, 6. Overall feasible to implement using existing resources  7, 13. Cost of data collection was £4.60 per child  **Complexity, flexibility, manualisation, and time concerns**  2. Disorganised paper records; incomplete data; discrepancies in data 3. 3/4 nurses said that it was time-consuming to extract data: mean total time per child screened was 22 minutes (11 min from school records, 11 from London Child Health Computer System) |
| Fox (2013)  Depression and suicide risk | Researcher-designed survey  Parents | **Intervention fit**  9, 14. 84-89% of parents supported school-based depression and suicide screening & education.  9, 14. Support for suicide screening associated with fewer stigmatising beliefs about suicide and depression (OR 0.03, CI (0.01-0.10); more likely among parents of white ethnicity compared to non-white ethnicity (85.8% vs. 64.5%, p=0.002) and those with history of mental health disorder (91.4% vs. 82.5%, p=0.016)  9, 14. Support of depression screening associated with greater knowledge (OR 8.48, CI (1.30-55.21)) and fewer stigmatising beliefs about suicide and depression (0.03, CI (0.01-0.12)); more likely among white than non-white parents (84.7% vs. 71.0%, p=0.045) and those with history of mental health disorder (89.0% vs. 82.2%, p = 0.040) |
| Gilmore (2004)  Behavioural problems | Evaluation forms  Focus groups  Teachers | **Intervention fit**  14. Proactive screening meeting was seen as useful  14. Both checklists were seen as useful and no major alterations were suggested  **Complexity, flexibility, manualisation, and time concerns**  2. Proactive screening meeting was seen easy to set up  3. Time constraints were a concern (though not differentiated between screening and intervention) |
| Gould (2005)  Suicide risk | Authors’ observations  NA | **Adverse events**  8. Levels of distress did not vary depending on exposure to questions about suicide either immediately after the screening (exposed POMS-A =5.5; control POMS-A=5.1) or two days later (exposed POMS-A =4.3; control POMS-A=3.9); students exposed to questions no more likely than control students to report suicidal ideation (4.7% vs. 3.9%, respectively); high risk students in exposed group were not more distressed or more likely to report suicidal ideation than high risk students in control group. |
| Hallfors, Brodish (2006)  Suicide risk | Authors’ observations  NA | **Intervention fit**  9, 11. Upon receiving feedback, programme was modified to prioritise students based on risk such that counsellors could first help those at greatest risk.  9. The screener was found to result in a high number of false positives. Authors made suggestions for using the tool only for the highest risk groups. 14. Opinions were mixed on the importance of screening. Most personnel designated to conduct follow-up interviews were supportive of the programme, but many staff and administrators were hesitant to try a new programme. Many staff did not feel the screening had great value because most students were at low to moderate risk, and it was difficult to screen persistently truant students.  **Cost & resource implications**  1. All staff were offered training, but many declined, believing their professional training was sufficient. 3, 4, 5. The number of students recommended for follow-up overwhelmed school staff, and schools had to designate specific teams to assess identified students. One school required additional staff. Schools required continued monitoring and reminders to follow-up in the post-screening period. Some schools were expected to assess 10-25 students per week, which was considered unrealistic.  **Complexity, flexibility, manualisation, and time concerns**  2. Only a few schools were able to comply fully with the protocols for the programme.  3, 4, 5. The number of students recommended for follow-up overwhelmed school staff, and schools had to designate specific teams to assess identified students. One school required additional staff. Schools required continued monitoring and reminders to follow-up in the post-screening period. Some schools were expected to assess 10-25 students per week, which was considered unrealistic.  9, 11. Upon receiving feedback, programme was modified to prioritise students based on risk such that counsellors could first help those at greatest risk. |
| Hallfors, Cho (2006)  Substance use | Authors’ observations  NA | **Cost & resource implications**  2, 6. School record data were readily available and cut-points were easy to use, and worked well with the teacher referral system.  **Complexity, flexibility, manualisation, and time concerns**  2, 6. School record data were readily available and cut-points were easy to use, and worked well with the teacher referral system. |
| Hallfors (2000)  Substance (alcohol, tobacco & other drugs) use | Authors' observations  NA | **Intervention fit**  9. Teachers who preferred computer assisted self-interviewing (CASI) said it was more applicable to students than paper and pencil  9. Students preferred CASI (in Vallejo, 60% preferred CASI - 33% expressed no preference)  14. Teachers' perceptions of formats were directly related to school readiness; 6/15 teachers preferred CASI, 5/15 preferred paper and pencil  **Cost & resource implications**  2, 6. CASI difficult to administer because of varied quantity/quality of computer resources  3, 5, 6. Teachers who preferred paper and pencil cited limited resources and time intensity/interruption of class time as key issues  5. One school not willing to allocate staff time to prepare & maintain computers  6. Schools had ratios of 20:1 to 100:1 computers: students (one school did not have computers); researchers had to bring additional computers to 3 schools to complete screening  6. Limited room capacity to conduct screening  **Complexity, flexibility, manualisation, and time concerns**  2, 3. Difficulty getting students to complete the screening (e.g. difficulty locating them/getting them to attend, persuading teachers to release students' time)  2, 3. Teachers who preferred CASI said it was easier to set-up and less time-consuming than paper and pencil screening  2, 3. Data collection was much less cumbersome for CASI - data immediately available & aggregated into a database; pencil and paper data collection took 5 weeks - 4 months to collect  2, 6. CASI difficult to administer because of varied quantity/quality of computer resources  3, 5, 6. Teachers who preferred paper and pencil cited limited resources and time |
| Kirk (2014)  Behavioural and socioemotional problems | Researcher-designed survey to assess teachers' perceptions of the BASC-2 BESS and the screening process with 5-point Likert scale where 1 = strongly disagree and 5 = strongly agree  Focus groups to understand teachers' perceptions of internalising and externalising behaviours and the role of teachers in screening  Teachers | **Intervention fit**  9. Information received from the screening was useful for instructional practices (M = 3.42, SD=1.121), understanding student needs (M =3.42, SD = 1.21), and talking with parents (M = 3.72, D = 1.52)  14. Time spent completing the BASC-2 BESS was useful (M = 3.54, SD = 1.13)  **Complexity, flexibility, manualisation, and time concerns**  2. Getting consent from parents was challenging  3. One teacher found the process time-consuming and not a good use of time |
| Lyon (2016)  Depression | System dynamics modelling  NA | **Cost & resource implications**  5. Screening may lead to a backlog of students accessing mental health services when capacity is limited & increases workload - increasing number of mental health professionals to 3 per 1000 students might be sufficient to address capacity problems  5. Employment of short-term mental health providers may help alleviate the service burden by performing assessments & lead to reduced waiting times following positive screens; may be more favourable for schools  5. Establishment of a queue for students in need of further assessment after the MFQ is near-immediate |
| McManus (2009)  Behavioural and socioemotional problems | Parents & Teachers: Participant Satisfaction Survey  Teachers: ASQ:SE Coaching Questionnaire  Parents, teachers | **Intervention fit**  2, 9. 91% of parents believed the questionnaire was easy to understand at 80% completed it without assistance; 3% of parents made suggestions to make the ASQ:SE clearer 9. 8/8 teachers believed ASQ:SE was age-appropriate; 8/8 teachers said the language was easily understandable; 4/8 teachers said the questions were culturally appropriate, 4/8 said they were somewhat appropriate 9. 93% of parents believed the questions were age-appropriate  9. 61% of 141 parents found the questionnaire interesting & 62% said it helped them think about their child’s social-emotional development (14% did not learn anything new)  14. 6/8 teachers would use the ASQ:SE in the future, 1/8 might use it, and 1/8 would not use it  **Complexity, flexibility, manualisation, and time concerns**  2. 8/8 teachers found the questionnaire easy to implement; 8/8 teachers said that either most parents completed the questionnaires independently or that they had only to answer a few questions 2, 14. 99% of parents did not believe the questionnaire was too long or that it was a waste of time  2, 9. 91% of parents believed the questionnaire was easy to understand at 80% completed it without assistance; 3% of parents made suggestions to make the ASQ:SE more clear  3. 2/8 teachers found it to take <10 minutes to complete survey, 5/8 said 10-20 minutes, and 1/8 said 20-30 minutes |
| Nemeroff (2008)  Behavioural and socioemotional problems | Authors’ observations  NA | **Intervention fit**  9. Counsellors/MH staff believed screening helped high-risk children get support  **Complexity, flexibility, manualisation, and time concerns**  2. Counsellors/MH staff felt intimidated by computerised assessments and preferred previously used assessments; some staff did not use the DISC even once over the study period 3. Counsellors/MH staff had limited time for MH assessments; data suggest it takes a year to routinely adopt the screening process 11. Counsellors/MH staff valued the flexibility of the DISC (e.g. to use for all or some students), and listed this as a main reason for its overall feasibility |
| Poulsen (2015)  Behavioural and socioemotional problems (post-disaster) | Postdisaster Screening Evaluation (measures satisfaction with screening programme)  Parents | **Intervention fit**  9, 14. 99.2% ‘very’ or ‘mostly’ satisfied with the programme overall (mean = 21.55/24); parents were more satisfied when results indicated ‘no’ or ‘minimal’ distress (compared with ‘moderate’ or ‘severe’), when their pre-screening concern was aligned with the screening result, and when they agreed with the result |
| Robinson (2011)  Suicide risk | Authors’ observations    Suicide Prevention Program Rating Profile (SPPRP): Survey rated on a 6 point Likert scale of 1-6 where 1=strongly disagree and 6=strongly agree  Students, parents, school staff | **Intervention fit**  9, 14. Authors concluded students, parents, and school staff believed the screening was acceptable and appropriate (Acceptability Factors = 31.05/45, 36.07/45, and 36.78, respectively) and that it was not overly intrusive (Intrusiveness Factors = 9.04/15, 10.79/15, and 10.74/15, respectively)  9, 14. Only 37% of the 30% of students who answered ‘yes’ to questions of low mood, deliberate self-harm, or suicidal behaviour found the screening programme helpful  9, 14. Over 70% of students found the screening questions ‘moderately’ or ‘very’ worthwhile, but at-risk students were less likely to find it worthwhile  **Complexity, flexibility, manualisation, and time concerns**  3. Authors concluded students, parents, and school staff believed screening was not overly time intrusive (Time Demands Factors = 6.84/10, 7.39/10, and 6.67/10, respectively)  **Adverse events**  8. School staff unaware of any distress caused to students  8. Study found no iatrogenic effect of screening: students exposed to screening did not have higher levels of distress: 8.9% of students reported finding the questions "moderately" or "very" distressing; at-risk students were more likely to find the questionnaire distressing compared to their peers |
| Romer (2012)  Risk for behavioural or socioemotional problems | Researcher-developed social validity assessment: rated on a 5-point Likert scale where 1=negative and 5=positive  Teachers, students | **Intervention fit**  14. Teachers believed screening was an acceptable use of class time (M = 3.93, mode = 4)  14. Students believed it was important for teachers and adults to help students with socioemotional problems (M = 3.788, mode=4) |
| Shortt (2006)  Risk for mental health difficulties | Pre- and post-surveys  School staff, child and adolescent mental health services staff (CAMHS) | **Intervention fit**  14. School staff believed that the programme was beneficial for students and there was a high level of satisfaction with outcomes (82% ‘very satisfied’)  14. CAMHS reported high satisfaction with student outcomes (5.12 on 6-point scale) |
| Vander Stoep (2005)  Behavioural and socioemotional problems | Authors’ observations  NA | **Cost & resource implications**  5. Screening required teams of 9-12 staff to screen all students; screening programme relied on full-time study staff and part-time temporary staff 6, 7, 13. Cost of screening implementation and 1:1 follow-up was calculated at US$9-15 per student (varied by school size and positive screening result prevalence). Costs included personnel costs, translators, questionnaire-associated expenses, recruitment expenses, supplies, incentives, thank you gifts for schools  **Complexity, flexibility, manualisation, and time concerns**  3. Screening was easily completed in a single 50-minute class period (with first 25 minutes used for organisational tasks); all students were screened in 1 to 1.5 days. |
| Walker (1994)  Behavioural and socioemotional problems | Consumer satisfaction surveys comparing traditional identification procedures with the Systematic Screening for Behavior Disorders (SSBD)  Teachers and school psychologists/resource teachers | **Intervention fit**  9, 14. Teachers from 2 of 3 schools would recommend SSBD to other schools  9, 14. 100% of 8 school psychologists/resource teachers found SSBD helpful and 75% would recommend to other teachers/resource teams  **Cost & resource implications**  7, 13. The cost of the screening was US$175.00, representing a cost difference from standard procedure of US$7.00  **Complexity, flexibility, manualisation, and time concerns**  3. The time per student required to complete SSBD procedures was 6.43 hours, which is 25 minutes less than the traditional assessment procedures (6.85 hours per student) |
| **STAFF IN-SERVICE TRAINING** | | |
| Nadeem (2016)  Suicide risk | Focus group and interviews  Teachers, administrators, and other school personnel | **Intervention fit** 9, 14. Some thought schools had too much responsibility already in supporting transition from middle to high school  **Cost & resource implications**  1. School staff indicated that staff should be better trained to support, educate, and involve parents in their child's mental health 5, 6. Mental health resources and counselling services on school campus were incredibly important in providing support; many parents did not have access to outside services  **Complexity, flexibility, manualisation, and time concerns**  2. Staff reported frustration with communication with parents, who often could not be reached after identification of suicidal ideation 11. Schools adapted programme by providing 'safe space' support for parents, or making home visits |
| Sayal (2006)  ADHD | Feedback forms  Teachers | **Intervention fit**  9. 92% of teachers felt they are well-placed to identify ADHD; 87% felt more informed about ADHD after the training and 81% felt more confident about identification  14. 98% of teachers believed that identifying children with ADHD was relevant to their work and would be helpful to both teachers and children |
| **CURRICULUM-BASED MODEL** | | |
| Kalafat (1994)  Suicide risk | Survey  Students | **Intervention fit**  9. 43% of students rated classes as helpful (53% as neutral)  9. Suicide awareness classes rated more favourably than regular health classes (44% interesting/very interesting vs. 32% interesting/very interesting)  9. 39% of students indicated they learned a lot/more than average (38% rated as average; 23% rated as low in learning)  9. 66% of students rated class material as 'about right' (14% rated as 'too advanced'; 20% as 'too elementary')  9. 81% of students thought other students should participate in the programme  **Adverse events**  8. 3% of students rated classes as upsetting |
| **COMPARATIVE – UNIVERSAL SCREENING VS. STAFF IN-SERVICE TRAINING VS. CURRICULUM-BASED** | | |
| Eckert (2006)  Suicide risk | Suicide Prevention Program Rating Profile (SPPRP): Survey rated on a 6 point Likert scale of 1-6 where 1=strongly disagree and 6=strongly agree  University students | **Intervention fit**  3, 9. Females rated curriculum-based models as significantly more acceptable, less intrusive, and less time-consuming than did males  9. Females rated staff in-service training as more acceptable than did males  9. No other statistically significant differences found for any programme  **Complexity, flexibility, manualisation, and time concerns**  3, 9. Females rated curriculum-based models as significantly more acceptable, less intrusive, and less time-consuming than did males |
| Eckert (2003)  Curriculum-based, staff in-service training, and universal screening | Suicide Prevention Program Rating Profile (SPPRP): Survey rated on a 6 point Likert scale of 1-6 where 1=strongly disagree and 6=strongly agree  School psychologists | **Intervention fit**  Universal screening:  9, 14. School-wide screening significantly less acceptable and more intrusive than staff in-service training or curriculum-based models  9, 14. Mean score for Acceptability Factor: 30.27 (SD 10.81); mean score for Intrusiveness Factor: 16.96 (SD 4.77)  9, 14. Mean score of 3.41 (SD 1.49) for the statement ‘This would be an acceptable suicide prevention program’  9. Mean score of 3.25 (SD 1.36) for the statement ‘Most school psychologists would find this suicide prevention program appropriate’  14. Mean score of 3.80 (SD 1.25) for the statement 'Overall, this program would be beneficial for students'  Staff in-service training:  9, 14. Mean score for Acceptability Factor: 38.11 (SD 5.16); mean score for Intrusiveness Factor: 14.33 (SD 5.16)  9, 14. Mean score of 4.54 (SD 1.22) for the statement ‘This would be an acceptable suicide prevention program’  9. Mean score of 4.37 (SD 1.16) for the statement ‘Most school psychologists would find this suicide prevention program appropriate’  14. Mean score of 4.59 (SD 1.03) for the statement 'Overall, this program would be beneficial for students'  Curriculum-based models:  9, 14. Mean score of 4.40 (SD 1.20) for the statement ‘This would be an acceptable suicide prevention program’  9, 14. Mean score for Acceptability Factor: 37.69 (SD 9.20); mean score for Intrusiveness Factor: 13.87 (SD 4.53)  9. Mean score of 4.40 (SD 0.98) for the statement ‘Most school psychologists would find this suicide prevention program appropriate’  14. Mean score of 4.48 (SD 1.02) for the statement 'Overall, this program would be beneficial for students'  **Complexity, flexibility, manualisation, and time concerns**  Universal screening: 3. Mean score of 2.83 (SD 1.25) (reverse scored) for statement 'This program was overly intrusive into the teacher's or staff member's time'; mean score of 2.55 (SD 1.10) (reverse scored) for statement 'This program was not intrusive into the student's or classroom instruction time'  Staff in-service training: 3. Mean scores of 2.82 (SD 1.39) (reverse scored) and 2.49 (SD 1.06) (reverse scored) for above statements, respectively.  Curriculum-based models: 3. Mean scores of 2.70 (SD 1.09) (reverse scored) and 2.57 (SD 1.08) (reverse scored) for above statements, respectively. |
| Miller (1999)  Curriculum-based, staff in-service training, and universal screening | Suicide Prevention Program Rating Profile (SPPRP): Survey rated on a 6 point Likert scale of 1-6 where 1=strongly disagree and 6=strongly agree  School principals | **Intervention fit**  Universal screening:  9, 14. Mean score of 3.86 (SD 1.30) for the statement ‘This would be an acceptable suicide prevention program’  9. Mean score of 3.91 (SD 1.22) for the statement ‘Most principals would find this suicide prevention program appropriate’; mean score of 3.82 (SD 1.03) for the statement 'This program would be appropriate for a variety of students'  14. Mean score of 3.80 (SD 1.12) for the statement 'I would find these prevention programs helpful for preventing adolescent suicides in my school'; mean score of 3.78 (SD 1.16) for the statement 'Overall, this program would be beneficial for students'  Staff in-service training:  9, 14. Mean score of 4.66 (SD 0.92) for the statement ‘This would be an acceptable suicide prevention program’  9. Mean score of 4.48 (SD 0.92) for the statement ‘Most principals would find this suicide prevention program appropriate’; mean score of 4.35 (SD 0.99) for the statement 'This program would be appropriate for a variety of students.'  14. Mean score of 4.32 (SD 0.92) for the above statement 'I would find these prevention programs helpful for preventing adolescent suicides in my school’; mean score of 4.56 (SD 0.76) for the statement 'Overall, this program would be beneficial for students'  Curriculum-based models:  9, 14. Mean score of 4.36 (SD 1.26) for the statement ‘This would be an acceptable suicide prevention program’  9. Mean score of 4.33 (SD 1.11) for the statement ‘Most principals would find this suicide prevention program appropriate’; mean score of 4.28 (SD 0.96) for the statement 'This program would be appropriate for a variety of students'  14. Mean score of 4.12 (SD 1.02) for the above statement 'I would find these prevention programs helpful for preventing adolescent suicides in my school’; mean score of 4.37 (SD 1.04) for the statement 'Overall, this program would be beneficial for students'  **Complexity, flexibility, manualisation, and time concerns**  Universal screening: 3. Mean score of 2.55 (SD 0.99) (reverse scored) for statement 'This program was not overly intrusive into the teacher's or staff member's time'; mean score of 2.56 (SD 0.90) (reverse scored) for statement 'This program was not overly intrusive into the student's or classroom instruction time.' This model was rated the most intrusive into student time.  Staff in-service training: 3. Mean scores of 2.50 (SD 1.05) (reverse scored) and 2.61 (SD 1.17) (reverse scored) for above statements, respectively. This model was rated the most intrusive into staff time.  Curriculum-based models: 3. Mean scores of 2.85 (SD 1.20) (reverse scored) and 2.85 (SD 1.19) (reverse scored) for above statements, respectively. This model was rated the least intrusive into student in teacher time. |
| Scherff (2005)  Curriculum-based, staff in-service training, and universal screening | Suicide Prevention Program Rating Profile (SPPRP): Survey rated on a 6 point Likert scale of 1-6 where 1=strongly disagree and 6=strongly agree  School superintendents | **Intervention fit**  Universal screening:  9, 14. School-wide screening significantly less acceptable and more intrusive than staff in-service training or curriculum-based models  9, 14. Mean score for Acceptability Factor: 24.87 (SD 7.36); mean score for Intrusiveness Factor: 6.38 (SD 2.19)  9, 14. Mean score of 3.66 (SD 1.17) for the statement ‘This would be an acceptable suicide prevention program’  9. Mean score of 3.59 (SD 1.09) for the statement ‘Most high school students would find this suicide prevention program appropriate’  14. Mean score of 3.62 (SD 1.15) for the statement 'Overall, this program would be beneficial for students.'  Staff in-service training:  9, 14. Mean score for Acceptability Factor: 28.24 (SD 7.13); mean score for Intrusiveness Factor: 7.36 (SD 2.38)  9, 14. Mean score of 4.19 (SD 1.41) for the statement ‘This would be an acceptable suicide prevention program’  9. Mean score of 4.00 (SD 1.14) for the statement ‘Most high school students would find this suicide prevention program appropriate’  14. Mean score of 4.21 (SD 1.14) (reverse scored) for the statement ‘Overall, this program would be beneficial for students’  Curriculum-based models:  9, 14. Mean score for Acceptability Factor: 28.91 (SD 6.25); mean score for Intrusiveness Factor: 7.19 (SD 1.96)  9, 14. Mean score of 4.37 (SD 0.95) for the statement ‘This would be an acceptable suicide prevention program’  9. Mean score of 4.15 (SD 1.08) for the statement ‘Most high school students would find this suicide prevention program appropriate’  14. Mean score of 4.27 (SD 0.90) (reverse scored) for the statement ‘Most high school students would find this suicide prevention program appropriate’  **Complexity, flexibility, manualisation, and time concerns**  Universal screening: 3. Mean scores of 3.97 (SD 1.06) (reverse scored) for statement 'This program was overly intrusive into the teacher's or staff member's time'; mean score of 3.91 (SD 1.12) (reverse scored) for statement 'This program was not intrusive into the student's or classroom instruction time.'  Staff in-service training: 3. Mean scores of 3.86 (SD 1.37) (reverse scored) and 4.12 (SD 1.09) (reverse scored) for above statements.  Curriculum-based models: 3. Mean scores of 3.77 (SD 1.16) (reverse scored) and 4.03 (SD 1.02) (reverse scored) for above statements, respectively. |
| Whitney (2011)  Curriculum-based, staff in-service training, and universal screening | Semi-structured interviews  School principals | **Intervention fit**  9, 14. All but one (middle school) principal believed that staff in-service training was the most acceptable approach (the middle school principal preferred the curriculum-based model)  Universal screening:  9. Concerns students would not be truthful in their responses 9, 14. Concerns about resistance from parents; concerns of whether this is a school responsibility  Staff in-service training:  9, 14. Concerns about lack of teacher buy-in on an annual basis  Curriculum-based models:  9. Primary school principal doubted the appropriateness for younger students 9, 14. Concerns about lack of teacher buy-in and possible parental objections  **Cost & resource implications**  Universal screening:  5, 6. Screening would require a lot of additional resources for implementation  Staff in-service training:  1. Training would increase staff awareness of suicidal behaviours  **Complexity, flexibility, manualisation, and time concerns**  Staff in-service training:  2. Easy to implement in comparison to other models  2, 10. Uniformity is key in this model  Curriculum-based models:  2. Easy to implement and deliverable to all students 3. Concerns about relatively short time frame; concerns about competing with other subjects 10. Standardised approach across students and schools |

NA = not applicable

K-12 = kindergarten through 12^th^ grade (USA)

MH = mental health
