# Supplementary material for: Feasibility of School-Based Identification of Children and Adolescents Experiencing, or At-risk of Developing, Mental Health Difficulties: a Systematic Review
Source: Prev Sci. 2020 Feb 15;21(5):581–603. doi: 10.1007/s11121-020-01095-6 (PMC7305254; doi:10.1007/s11121-020-01095-6)
Supplement: Supplementary file 5 — (DOCX 20.7 kb) [file 11121_2020_1095_MOESM5_ESM.docx]

**Supplementary 4. Aspects of feasibility addressed by included studies**

|  | **Barry (2016)** | **Bruhn (2014)** | **Chartier (2008)** | **Chatterji (2004)** | **Curtis (2014)** | **Davis (2014)** | **Donohue (2015)** | **D'Souza (2005)** | **Eckert (2006)** | **Eckert (2003)** | **Edmunds (2005)** | **Fox (2013)** | **Gilmore (2004)** | **Gould (2005)** | **Hallfors, Brodish (2006)** | **Hallfors, Cho (2006)** | **Hallfors (2000)** | **Kalafat (1994)** | **Kirk (2014)** | **Lyon (2016)** | **McManus (2009)** | **Miller (1999)** | **Nadeem (2016)** | **Nemeroff (2008)** | **Poulsen (2015)** | **Robinson (2011)** | **Romer (2012)** | **Sayal (2006)** | **Scherff (2005)** | **Shortt (2014)** | **Vander Stoep (2005)** | **Walker (1994)** | **Whitney (2011)** |
| --- | --- | --- | --- | --- | --- | --- | --- | --- | --- | --- | --- | --- | --- | --- | --- | --- | --- | --- | --- | --- | --- | --- | --- | --- | --- | --- | --- | --- | --- | --- | --- | --- | --- |
| **Staff training** | x |  |  |  |  |  |  |  |  |  |  |  |  |  | x |  |  |  |  |  |  |  | x |  |  |  |  |  |  |  |  |  | x |
| **Intervention complexity** | x | x | x |  |  | x | x |  |  |  | x |  | x |  | x | x | x |  | x |  | x |  | x | x |  |  |  |  |  |  |  |  | x |
| **Time consuming** | x |  |  |  | x | x | x | x | x | x | x |  | x |  | x |  | x |  | x |  | x | x |  | x |  | x |  |  | x |  | x | x | x |
| **Ongoing supervision** |  |  |  |  |  |  |  | x |  |  |  |  |  |  | x |  |  |  |  |  |  |  |  |  |  |  |  |  |  |  |  |  |  |
| **Additional human resources** |  | x |  | x |  |  | x | x |  |  |  |  |  |  | x |  | x |  |  | x |  |  | x |  |  |  |  |  |  |  | x |  | x |
| **Additional material resources** |  | x |  | x |  |  | x |  |  |  |  |  |  |  |  | x | x |  |  |  |  |  | x |  |  |  |  |  |  |  | x |  | x |
| **Costly set up** |  | x |  | x |  |  |  |  |  |  | x |  |  |  |  |  |  |  |  |  |  |  |  |  |  |  |  |  |  |  | x | x |  |
| **Adverse events** |  |  |  |  |  |  |  |  |  |  |  |  |  | x |  |  |  | x |  |  |  |  |  |  |  | x |  |  |  |  |  |  |  |
| **Applicable to population of interest** | x |  |  |  |  | x | x | x | x | x |  | x | x |  | x |  | x | x | x |  | x | x | x | x | x | x |  | x | x |  |  | x | x |
| **Manualised** |  |  |  |  |  |  |  |  |  |  |  |  |  |  |  |  |  |  |  |  |  |  |  |  |  |  |  |  |  |  |  |  | x |
| **Flexibility** |  |  |  |  | x |  |  | x |  |  |  |  |  |  | x |  |  |  |  |  |  |  | x | x |  |  |  |  |  |  |  |  |  |
| **Cost saving** |  |  |  | x |  |  |  |  |  |  | x |  |  |  |  |  |  |  |  |  |  |  |  |  |  |  |  |  |  |  | x | x |  |
| **Matches prioritised goals** | x | x |  |  |  | x |  | x |  | x |  | x |  |  | x |  | x |  | x |  | x | x | x |  | x |  | x | x | x | x |  | x | x |
