# Supplementary material for: Feasibility of School-Based Identification of Children and Adolescents Experiencing, or At-risk of Developing, Mental Health Difficulties: a Systematic Review
Source: Prev Sci. 2020 Feb 15;21(5):581–603. doi: 10.1007/s11121-020-01095-6 (PMC7305254; doi:10.1007/s11121-020-01095-6)
Supplement: Supplementary file 6 — (DOCX 21 kb) [file 11121_2020_1095_MOESM6_ESM.docx]

**Universal (school-wide) screening:** School-wide screening refers to the ***systematic evaluation of* all *students in a school for risk for poor mental health outcomes, as identified by the presence of psychosocial risk, symptomatology, or functional impairment*.** Many school-wide screening programmes are multi-gated, meaning that students are first universally screened using standardised tools, and then those who score above a pre-defined cut-off score in the first stage are referred onward to clinical interviews or further evaluation. Measurement tools may be completed by students, parents, teachers, or a combination thereof. In countries such as the UK, where the vast majority of children attend school, school-wide screening may also be viewed as universal screening for CYP.

*For an exemplary programme, see Husky and colleagues’ universal screening programme for identifying MHD (Husky, Sheridan, McGuire, & Olfson, 2011). In this programme, students complete an abbreviated form of the Diagnostic Interview Schedule for Children IV. Following the screening, students meet one-on-one with a staff member to discuss results. Students who score positively on the self-report measure or who ask for help in the discussion afterwards attend a clinical interview after which they receive recommendations and/or referrals.*

**Selective screening:** The term ‘selective (or targeted) screening’ applies when ***all students within a particular high-risk group are screened***. Effectively, selective screening aims to target groups with higher prevalence of MHD so as to increase the positive predictive value of the tool and thereby limit the number of false positives results. With regard to mental health screening in CYP, high-risk groups may include students with histories of behavioural incidents, office discipline referrals, or high number of absences, as well as new students (Shortt, Fealy, & Toumbourou, 2006).

*For an exemplary programme, see Hallfors and colleagues’ selective screening programme for suicide risk (Hallfors et al., 2006a). This programme used the Suicide Risk Screen to screen a group of ‘high risk’ students. These students were part of Reconnecting Youth, a programme for students who exhibit risky behaviours (e.g. substance use) that aims to enhance personal and social protective skills. These students, therefore, were deemed to be at higher risk than their peers for suicide risk. Following screening, school staff contacted parents and provided referrals when necessary.*

**Staff in-service training:** Staff in-service training (also known as the school gatekeeper model) refers to ***trainings presented to school staff (usually by mental health professionals) that aim to increase knowledge of a certain condition and build capacity to recognise and refer students experiencing difficulties***. Staff in-service training may aim to educate *all staff* in a school or only a sub-set (e.g. teachers and teaching assistants). These trainings often have a broader focus than just identifying MHD, and may provide knowledge and skills related to the entire pathway, from identification to response to further referral.

*For an exemplary programme, see Sayal and colleagues’ teacher training programme for identifying ADHD (Sayal, Hornsey, Warren, MacDiarmid, & Taylor, 2006).* *This programme consisted of a 45-minute training delivered to teachers by a child and adolescent psychiatrist and a research worker. The session included information about signs and symptoms of ADHD, its role as a risk factor, how it presents in students, possible associated outcomes, diagnoses/medication, and classroom management strategies.*

**Curriculum-based models:** Curriculum-based models of MHD identification are targeted directly at students. Curriculum-based models ***rely on students to identify themselves and each other as at-risk after attending classes on the condition of interest.*** In this model, students are provided information and skills to help them recognise warning signs, support those experiencing difficulties, determine next steps, and communicate concerns to the appropriate adults. Materials may be presented by mental health professionals or school staff (e.g. teachers). As with the staff in-service training model, these programmes often go beyond simply identifying MHD. For example, they may also include objectives of promoting psychological wellbeing, reducing stigma surrounding MHD, or improving mental health literacy more generally.

*For an exemplary programme, see the Signs of Suicide (SOS) prevention programme (Aseltine Jr & DeMartino, 2004), which combined a curriculum-based approach with a brief screening. The curriculum aimed to raise awareness of suicide and related issues. Students learned about how to recognise signs of suicide in themselves and others, support peers with identified risk, and speak with an adult about their concerns.*

Panel 1. Further explanation and examples of school-based MHD identification models.

Aseltine Jr, R. H., & DeMartino, R. (2004). An outcome evaluation of the SOS suicide prevention program. *American Journal of Public Health, 94*(3), 446-451.

Hallfors, D., Brodish, P. H., Khatapoush, S., Sanchez, V., Cho, H., & Steckler, A. (2006a). Feasibility of screening adolescents for suicide risk in “real-world” high school settings. *American Journal of Public Health, 96*(2), 282-287.

Husky, M. M., Sheridan, M., McGuire, L., & Olfson, M. (2011). Mental health screening and follow-up care in public high schools. *Journal of the American Academy of Child & Adolescent Psychiatry, 50*(9), 881-891.

Sayal, K., Hornsey, H., Warren, S., MacDiarmid, F., & Taylor, E. (2006). Identification of children at risk of Attention Deficit/Hyperactivity Disorder. *Social psychiatry and psychiatric epidemiology, 41*(10), 806-813.

Shortt, A. L., Fealy, S., & Toumbourou, J. W. (2006). The mental health Risk Assessment and Management Process (RAMP) for schools: II. Process evaluation. *Australian E-journal for the Advancement of Mental Health, 5*(3), 295-306.
