# Supplementary material for: Feasibility of School-Based Identification of Children and Adolescents Experiencing, or At-risk of Developing, Mental Health Difficulties: a Systematic Review
Source: Prev Sci. 2020 Feb 15;21(5):581–603. doi: 10.1007/s11121-020-01095-6 (PMC7305254; doi:10.1007/s11121-020-01095-6)
Supplement: Supplementary file 7 — (DOCX 162 bytes) [file 11121_2020_1095_MOESM7_ESM.docx]

**Table 1. Characteristics of included studies**

| **1^st^ author (year); country**  **Condition** | **Study design^1^** | **Study aims** | **School level(s)**  **Informants** | **Identification measure(s)** | **Study description**  **Follow-up mechanism for students identified as having MHD/risk for MHD** | **Sample characteristics** | **Percentage of students identified as having MHD/risk for MHD** |
| --- | --- | --- | --- | --- | --- | --- | --- |
| **UNIVERSAL & SELECTIVE SCREENING** | | | | | | | |
| **Barry (2016); USA**  **ADHD** | Cross- sectional | To explore the feasibility of school-based identification of children at-risk for ADHD and the communication of results and recommendations to parents. | Elementary school  Teachers | Vanderbilt AD/HD Diagnostic Teacher Rating Scale (VADTRS)  School Intervention Questionnaire (SIQ) | (1) Teacher-report questionnaire (VADTRS); (2) teacher-report questionnaire (SIQ) for children identified as at-risk by the VADTRS  Results fed back to parents with recommendation to see primary care provider for further evaluation | Students: n = 5772 (1st-5th grade; mean age 8.7 years; 66.4% male) | 18.1% |
| **Bruhn (2008); USA**  **Behavioural and socioemotional problems** | Cross- sectional | To examine current screening practices and barriers to screening implementation in K-12 schools. | Elementary school Middle school High school  ND | NA | NA  NA | School- or district-level administrators: n = 454 (53.3% male; 67.6% district administrators, 22.2% principals, 2.2% counsellors, 3.1% school psychologists, 2.4% vice principals, 2.4% other) | NA |
| **Chartier (2008); USA**  **Behavioural and socioemotional problems** | Interrupted time series | To examine the difference in participation rates in a school-wide screening programme (the Developmental Pathways Screening Program) under passive versus active parental consent conditions. | Middle school  Students | Mood and Feelings Questionnaire (MFQ) | (1) Student-report questionnaire (MFQ); (2) students who scored above cut-off received clinical evaluation  Results fed back to parents with referrals made to school- and community-based services as appropriate | 2002-3:  Students: n = 1011 (6th grade; no further characteristics provided)  2003-4: Students: n = 1021 (6th grade; no further characteristics provided) | 2002-3: 13.7%  2003-4: 14.7% |
| **Chatterji (2004); USA**  **Anxiety, depression, substance use disorders** | Economic evaluation & pre-post design | To use cost-analysis methods in a real-world setting.  To estimate costs of a school-based mental health screening and treatment programme over two years of operation. | Middle school  Students | Diagnostic Interview for Children (DISC) Predictive Scale (DPS)  Children's Global Assessment Scale (CGAS) | (1) Paper and pencil DPS; (2) DISC by an interviewer for all students who indicated suicidal behaviour or significant mood, substance use, or anxiety problems. *NB: in Year 2, students completed the Voice DISC if they spoke English (interviewer for non-English speaking students) and only new students (mostly sixth graders) were screened*. In case of suicidal behaviours on DPS, students were screened by screening director and upon confirmation were seen by a psychiatrist (evaluation using CGAS). In case of depression, anxiety, or substance use disorders on DPS, students completed depression portion of DISC (if marked anxiety on DPS, then completed anxiety portion of DISC; same for substance use)  Results fed back to parents with referral to school-based mental health services for individual or group counselling (most common referral type) or addition to waiting list for school- or community-based services as appropriate. | Year 1  Students: n = 1155 (grades 6, 7, 8)  Year 2  Students: n = 453 (mostly grade 6) | ND (10.0% and 2.4% of screened students referred to treatment in Years 1 and 2, respectively) |
| **Curtis (2014); USA**  **Substance abuse** | Cross- sectional | To assess the feasibility and economic sustainability of conducting screening, brief motivational counselling intervention and referral to treatment (SBIRT) in two urban schools. | Middle school High school  Students | CRAFFT Screening Tool For Adolescent Substance Abuse (CRAFFT) | (1) Student-report interactive screening instrument (CRAFFT)  Students with “some risk” received a brief motivational interview and could receive recommendation for continued sessions. Students with “significant risk” had their results fed back to parents, were offered a brief intervention, and were referred to formal treatment as appropriate. | Students: n = 248 (6th-12th grade; n = 106 middle school, n = 135 high school; 47.1% male) | 42% (25% at “moderate” risk; 18% at “significant” risk) |
| **Davis (2012); USA**  **Behavioural and socioemotional problems** | Cross- sectional | To compare teacher nomination process with the BASC-2 Behavioral and Emotional Screening System (BESS) for the detection of students with emotional and behavioural disorders. | Middle school  Teachers | Teacher Nomination Form (TNF)  Behavior Assessment System for Children, Behavioral and Emotional Screening System (BASC-2 BESS) | (1) "First gate" nomination/ ranking of 10 students likely to have emotional and behavioural disorders (5 externalizing, 5 internalizing); (2) "second gate" teacher-report questionnaire (BASC-2 BESS) for top 5 ranked externalizing and top 5 ranked internalizing students  Results fed back to teachers | Students: n = 2323 (51.3% male; no further information provided)  Teachers: n = 59 (23% male; mean years teaching = 9.45 (School 1), 12.14 (School 2)) | ND  (*NB:* author treated each nomination as a *separate student*, even if multiple teachers nominated the same student. 74% of total *nominations* screened positive.) |
| **Donohue (2016); USA**  **Behavioural and socioemotional problems** | Cross- sectional | To evaluate the process and outcomes of a school counsellor-led universal screening programme in one school district. | Elementary school Middle school High school  Students | Behavior Assessment System for Children, Behavioral and Emotional Screening System (BASC-2 BESS) | (1) Student-report questionnaire (BASC-2 BESS)  Results fed back to parents (for at-risk students only) with information on available school-and community support. Further assessment (if necessary) and group- or individual-level counselling provided for at-risk students. Regular meetings between counsellors, teachers, administrators, and special education professionals to discuss & monitor students. | Students: n = 94 (Grades 3, 4, 6, 7, 9, 10; no further characteristics are provided) | 9-10% (across 2 years of screening) |
| **D'Souza (2005); USA**  **Eating disorders** | Mixed methods | To evaluate the implementation and effectiveness of the high school version of the National Eating Disorders Screening Program. | High school  Students | National Eating Disorders Screening Program screening form - includes Eating Attitudes Test (EAT-26) | (1) Student-report questionnaire (NEDSP questionnaire)  Results fed back to students with recommendation to see a clinician about eating disorder symptoms as appropriate. | Students: n = 1027 (9th-12th grade; mean age = 15.9 years; 42% male)  School staff: n = 4 (no further characteristics provided) | 30% of girls & 16% of boys met criteria for clinical evaluation |
| **Edmunds (2005); UK**  **Behavioural and socioemotional problems** | Cross- sectional | To examine the feasibility of the Child Health Assessment at School Entry (CHASE) questionnaire.  To assess the acceptability of the questionnaire to parents, teachers, nurses.   To examine quality of obtained data and quantify the validity and reliability and of the questionnaire. | Primary school  Parents, school nurses | CHASE questionnaire comprising Strengths and Difficulties questionnaire (SDQ) and Child Health Questionnaire - Parent Form 28 (CHQ-PF28)  School nurse questionnaire using school health and education records | (1) Parent-report questionnaire (CHASE questionnaire) and school nurse questionnaire (in no defined order)  ND | Students: n = 278 (Year 1; no further characteristics provided)  School nurses: n = 7 (No further characteristics provided) | SDQ “borderline” score: 6.7%  SDQ “abnormal” score: 7.9% |
| **Fox (2012); USA**  **Depression and suicide risk** | Cross-sectional | To examine parental attitudes regarding school-based depression and suicide screening and education.  To identify predictors of positive perceptions of screening. | Elementary school  Middle school  High school  ND | NA | ND  NA | Parents: 511 (50.9% male; mean age = 44.9 years) | NA |
| **Gilmore (2004); New Zealand**  **Behavioural problems** | Mixed methods | To develop and evaluate a screening & intervention model at school entry. | Primary school  Teachers, keyworkers | Brief Behaviour Screening Checklist (5-item)  Behaviour Screening Checklist (28-item) | (1) Collaborative interview with teacher & keyworker (Proactive Screening Meeting; PSM); (2) teacher-report Brief Behaviour Screening Checklist for all children; (3) Behaviour Screening Checklist for children who are of concern during the PSM  Individual, group, class, and school-wide in-school interventions (implemented by teachers, with parent involvement). Some “home interventions”. | Students: n = 15 (No further characteristics provided) | ND |
| **Gould (2005); USA**  **Suicide risk** | RCT | To determine whether there is an iatrogenic effect of screening for suicide risk, i.e. does screening increase suicidal ideation or distress among a) general population of high school students, or b) high-risk population of students. | High school  Students | Profile of Mood States (POMS-A)  Suicidal Ideation Questionnaire (SIQ-JR)   Interim Depression and Suicidal Ideation  Beck Depression Inventory (BDI) and Drug Use Screening Inventory (DUSI) and Suicide Attempt History | 2 day screening strategy: (Day 1) all students completed POMS-A, BDI, DUSI, second POMS-A; students in experimental group additionally complete SIQ-JR and suicide attempt history; (Day 2) all students completed another POMS-A, an interim depression question, and 4 suicidal ideation measures (SIQ-JR, suicide attempt history, interim suicide item, BDI suicide item)  Further interview for students reporting serious distress, serious suicidal ideation, and suicide attempt. Referrals to treatment arranged as needed with parent involvement. | Students: n = 2342 (n = 1172 intervention and n = 1170 control) (9th-12th grade; mean age = 14.8 years) | ND (percentages of students screening positive for depression/  suicidal ideation are given only for the interim period between Days 1 and 2) |
| **Hallfors, Brodish (2006); USA**  **Suicide risk** | Cross- sectional | To assess the feasibility of a school- and population-based approach for suicide prevention in adolescents. | High school  Students | School records (i.e. a combination of absences and GPA) and teacher referral  High School Questionnaire (HSQ) audio computer assisted format  Suicide Risk Screen (SRS) | (1) HSQ used to determine which students were high risk: (a) in upper 25% of distribution of absences AND lower 50% of grade point average - GPA, OR (b) nominated by a teacher; (2) Student-report questionnaire (SRS) completed by typical and at-risk students  Follow-up interview conducted by school staff. Referrals made as necessary, and parents were given lists of community-based services. | Students: n = 1323 (9th-11th grade; 48.1% male); n = 393 typical students; n = 930 high risk students | 29% |
| **Hallfors, Cho (2006); USA**  **Substance use and related problems** | Case control | To examine the performance of a school-based screening method that uses school record data and teacher nomination. | High school  Students | School records (i.e. a combination of absences and GPA) and teacher referral  High School Questionnaire (HSQ) audio computer assisted format  Suicide Risk Screen (SRS) | (1) School records used to determine which students were high risk: (a) in upper 25% of distribution of absences AND lower 50% of grade point average - GPA, OR (b) nominated by a teacher; (2) student-report questionnaire (HSQ) to assess risk behaviours (SRS embedded)  ND | Students: n = 1323 (9th-11th grade; 48.1% male); n = 393 typical students; n = 930 high risk students | High risk group vs. “typical” group  Cigarette use: 24% vs. 9%  Alcohol use: 49% vs. 27%  Marijuana use: 31% vs. 15%  Other illegal drug use: 15% vs. 13%  Suicide risk: 34% vs. 18% |
| **Hallfors (2000); USA  Substance (alcohol, tobacco & other drugs) use** | Cross-sectional | To test whether computer-assisted self interviews (CASI) could be applied in public school settings to improve accuracy of substance use data.  To examine implementation, acceptability, and advantages of CASI. | Middle school High school  Students | Santa Barbara schools: Santa Barbara Student Substance Use Survey (adapted from the California State Substance Use Survey)  Vallejo schools: American Drug and Alcohol Survey; Prevention Planning Survey | (1) Student-report measure (either CASI or paper and pencil; measure varied by school district)  ND | Santa Barbara students: n = 1555 (grades 7, 9, 11)  Vallejo students: n = 1874 (grades 7, 9, 11) | ND |
| **Kirk (2014); USA**  **Behavioural and socioemotional problems** | Mixed methods | To compare three methods of screening for emotional and behavioural difficulties.  To explore teacher perspectives on the screening and examine screening acceptability. | Elementary school  Teachers | Behavior Assessment System for Children, Second Edition (BASC-2)  BASC-2 Behavioral and Emotional Screening System (BASC-2 BESS)  Teacher referral data and office discipline referrals (ODRs) | (1) Teacher-report questionnaires (BASC-2 BESS) for all students; BASC-2 for 5 randomly selected students; ODR and teacher referral data collected  Results fed back to parents and to teacher, with parents’ permission (for at-risk students only). “Follow-up support” provided by principal and school counsellor, where needed. | Students: n = 109 (Kindergarten-6th grade; 53% male; no further characteristics provided)  Teachers: n = 13 (8% male; mean years of teaching = 15.1 - range 2-29) | Screening with BASC-2 BESS: 21%  Teacher nomination: 25%  ODRs method: 5% |
| **Lyon (2016); USA**  **Depression** | Modelling study | To provide an example of the utility of system dynamics modelling.  To explore how system dynamics modelling can be used to inform decisions in school-based depression screening by identifying (1) components that can influence delivery & outcomes, (2) additional resource requirements, and (3) leverage points providing opportunity for addressing mental health needs. | High school  Students | Moods and Feelings Questionnaire (MFQ) | (1) Student-report questionnaire (MFQ); (2) assessment & referral by mental health provider  Model assumes mental health and non-mental health intervention options are available to identified students (model focuses on Interpersonal Therapy for Adolescent Depression as key mental health treatment) | Model assumes n = 1000 students | Model assumes 13.9% of students may score “high” for depression |
| **McManus (2009); USA**  **Behavioural and socioemotional problems** | Cross- sectional | To evaluate the implementation of a social-emotional screening program and how training, coaching, and monitoring of implementation affected teacher behaviour and child outcomes. | Elementary school  Teachers, parents | Ages & Stages Questionnaires: Social Emotional (ASQ:SE) | (1) Questionnaire (ASQ:SE) completed by teachers; (2) Teachers assist parents in the completion of ASQ:SE in home visits  Further assessment and individualised social-emotional/behavioural support for students identified as “at-risk” | Students: n = 141 (ages 3-5 years; 41% male)  Parents: n = 141 (76% biological mothers, 7% biological fathers, 17% other relatives) Teachers: n = 8 (3.5-29 years experience in Head Start programme) | ND |
| **Nemeroff (2008); USA**  **Behavioural and socioemotional problems** | Cross-sectional | To evaluate the feasibility of on-going school-based identification models for mental health problems. | Middle school Junior school High school  Students | Voice Diagnostic Interview Schedule for Children IV (DISC-IV)  Mental Health Tracking Form (MHTF) | (1) Counsellors had option to use Voice DISC-IV as part of student assessments (recording information in MHTF)  Results fed back to parent. Students identified as at-risk received recommendations for clinical evaluation with partnered clinics. | Students: n = 530 (aged 9-18 years; no further characteristics provided)  School counsellors and mental health staff: n = 41 (mean years counselling experience = 12.5; 19% male) | 72% of *students evaluated by counsellor* (*NB:* schools could choose to use programme as selective or universal screening) |
| **Poulsen (2015); Australia**  **Behavioural and socioemotional problems (post-disaster)** | Cross-sectional | To gauge parent satisfaction with post-disaster screening.  To determine if satisfaction was related to following through of screening recommendations.  To run subgroup analyses for these variables using exposure to disaster, parent concern, and demographic characteristics. | Primary school  Middle school  Secondary school  Parents | Postdisaster Screening Evaluation  UCLA Posttraumatic Stress Reaction Index (UCLA PTSD-RI)  Children's Depression Inventory - Short version (CDI-S)  Spence Children's Anxiety Scale (SCAS) | (1) Parent-report questionnaires (Postdisaster Screening Evaluation, UCLA PTSD-RI, CDI-S, SCAS)  Results fed back to parents with recommendations for further assessment/treatment as appropriate. | Students: n = 224 (aged 7-18 years; mean age = 11.0 years; 55% male)  Parents: n = 130 (13.1% male; no further characteristics provided | Moderate distress: 18.3%  Severe distress: 19.6% |
| **Robinson (2011); Australia**  **Suicide risk** | RCT | To implement an early identification programme for students at-risk for psychological distress, deliberate self-harm, or suicidal ideation. To determine whether there are associated iatrogenic effects. To assess the acceptability of the programme. | High school  Students | General Health Questionnaire (GHQ)  Profile of Mood States-A (POMS-A) | (1) Brief online student-report questionnaire completed over 2 days (students completed half on one day, half on the second). Half of the class completed the half with a screening question about distress/self-harm/suicidal ideation on the first day; half completed this half on the second day; (2) brief suicide/self-harm awareness workshop; (3) student-report questionnaires (GHQ, POMS-A)  At-risk students received clinical interviews with a member of the research team, along with referral to support as appropriate. | Students: n = 272 (Year 10; aged 14-16 years; all male) | 11.4% |
| **Romer (2012); USA**  **Risk for behavioural or socioemotional problems** | Cross-sectional | To evaluate the validity of the Social-Emotional Assets and Resilience Scales - (Student Short Forms) for the identification of middle school students at-risk for social/behavioural or mental health difficulties. | Middle school  Students, teachers | The Social-Emotional Assets and Resilience Scales - Short Form (SEARS-SF)  Youth Self-Report (YSR)  Behavioral and Emotional Screening System (BESS) teacher form | (1) Phase I: student-report questionnaire (SEARS-SF); (2) Phase II: 106 students (45 at risk and 61 not at-risk) completed YSR and SEARS-SF; teachers completed behaviour rating scales on participating students (BESS, SEARS-SF); student records used to collect ODRs, absences, and other information | Students: n = 1176 (characteristics reported on a school-by-school basis: 6-8th grade; ages 10-15; 43.6-51.5% male) | 21.7% |
| **Shortt (2006); Australia**  **Risk for mental health difficulties** | Pre-post | To evaluate screening programme in terms of teachers’ ability to identify at-risk students and intervene.  To explore the acceptability and feasibility of the RAMP programme. | Primary school  Secondary school  Teachers | RAMP screening form (no further description given) | (1) Systematic screening form (RAMP)  Individualised action plans for at-risk students, which may contain in-school support, school-family-community linkage, and/or referral to specific external mental health services. | Students: n = 422 primary school students (years 1-6); n = 61 secondary school students (years 7-10) No further characteristics provided. (*NB:* n = 483 students screened as part of the programme)  School staff: n = 34 primary school staff; n = 18 secondary school staff. No further characteristics provided. | Total screened positive ND  (n = 52 *newly identified* students of 483 screened) |
| **Vander Stoep (2005); USA**  **Behavioural and socioemotional problems** | Cross- sectional | To evaluate the feasibility, acceptability, and yield of the Developmental Pathways Screening Program (DPSP). | Middle school  Students | Developmental Pathways Screening Questionnaire (DPSQ), which contains items from Mood and Feelings Questionnaire (MFQ) and Youth Self Report (YSR) | (1) Student-report questionnaire (DPSQ); (2) school-based clinical assessment using DISC-IV for all students who scored positive for emotional distress  Results fed back to parents with referral as appropriate to interventions including academic tutoring, in-school counselling, and external mental health services. | Students: n = 861 (6th grade; 54.2% male) | 15.2% |
| **Walker (1994); USA**  **Behavioural and socioemotional problems** | Cross- sectional | To validate the results of the Systematic Screening for Behavior Disorders (SSBD) in an additional, non-norming site. | Elementary school  Teachers | Systematic Screening for Behavior Disorders (SSBD)  Social Skills Rating System (SSRS)  Office discipline referrals | (1) Stage 1: teacher nomination whereby teachers listed and ranked top 10 students exhibiting externalising behaviours and top 10 exhibiting internalising behaviours. (2) Stage 2: teacher-report Critical Events Index and Combined Frequency Index for adaptive/maladaptive behaviours. (3) Stage 3: direct observation of behaviours  Referral for further assessment as appropriate. | Students: n = 1468 (58 of which were previously diagnosed with behavioural disorder and served as comparison group; no further characteristics are provided)  Teachers and staff: n = 57  Special education resource teachers and psychologists: n = 8 | Stage 1: 32.4%  Stage 2: 15.3% (of the original sample) |
| **STAFF IN-SERVICE TRAINING** | | | | | | | |
| **Nadeem (2015); USA**  **Suicide risk** | Qualitative | To explore school personnel perspectives on parental involvement a district-wide suicide prevention programme. | Middle school  School personnel | ND | Youth Suicide Prevention Programme: (1) annual trainings for school-staff programme psychologist to develop skills to identify and refer at-risk students.  Students “in crisis” receive immediate support; schools contact parents and provide referrals to specialist services. Post-intervention phase includes developing in-school supports for students, following up with parents/external services, and facilitating school re-entry. | School staff: n = 45 (n = 7 mental health counsellors, n = 2 nurses, n = 26 teachers, n = 10 administrators; 42% male; mean years in education = 14) | ND |
| **Sayal (2006); UK**  **ADHD** | Cross-sectional | To examine the impact of an educational intervention for teachers to promote better recognition of ADHD. | Primary school  Teachers, parents | Strengths & Difficulties Questionnaire (SDQ) hyperactivity scale | (1) Teacher recognition of ADHD based on DSM-IV criteria; (2) SDQ screening (parent/teacher informants); (3) interactive teacher training including description of ADHD, presentation at school, ADHD as a risk factor, possible outcomes, importance/pervasiveness of symptoms, differential diagnoses/comorbidity, information about medication/classroom management strategies; (4) teacher recognition of ADHD  ND | Teachers: n = 96  Students: n = 2672 (mean age = 7.87, range 4-11 years; 50% male) | Teacher recognition at baseline: 3.2%  SDQ screening: 3-4%  Teacher recognition after training: 4.1%  (*NB:* estimates for “probable” ADHD) |
| **CURRICULUM-BASED MODEL** | | | | | | | |
| **Kalafat (1994); USA**  **Suicide risk** | Cross-sectional | To assess the efficacy of a high school suicide curriculum. | High school  Students | NA | (1) Education sessions for faculty, staff, and parents; training on procedure for responding to identified risk; establishment of links to community agencies; (2) half of students receive suicide awareness training in first marking period; half receive physical education classes (without suicide curriculum); (3) schedules reversed in the second marking period  Curriculum model included lesson plans for 3 40-50 min. participatory lessons:  1st lesson: information on suicide, attitudes toward suicide, tunnel thinking  2nd lesson: warning signs, roleplay with help-seeking focus  3rd lesson: video of consequences of not responding to peers, overview of school resources  ND | Students: n = 253 (grade 10; 57% male) | ND |
| **COMPARATIVE – UNIVERSAL SCREENING VS. STAFF IN-SERVICE TRAINING VS. CURRICULUM-BASED** | | | | | | | |
| **Eckert (2006); USA  Suicide risk** | Cross-sectional | To examine the acceptability to students of three school-based suicide prevention programmes. | High school  ND | NA | Curriculum based: (1) school psychologist to provide information on suicide (warning signs, incidence, etc.); (2) school psychologist to assess students identified as 'at-risk'  Staff in-service training: (1) staff receive 2-hr presentation on suicide prevention at beginning of school year; (2) school psychologist to assess students identified as 'at-risk'  School-wide screening: (1) self-report rating scale; (2) school psychologist to assess students identified as 'at-risk'  Results fed back to parents (for at-risk students only) with referral information | Students: n = 662 (freshmen in university; mean age = 17.99; 24.5% male) | NA |
| **Eckert (2003); USA**  **Suicide risk** | Cross- sectional | To explore school psychologists' perceptions of three different models of school-based suicide prevention programmes. | High school  ND | NA | Curriculum based: (1) school psychologist to provide information on suicide (warning signs, incidence, etc.); (2) school psychologist to assess students identified as 'at-risk'  Staff in-service training: (1) staff receive 2-hr presentation on suicide prevention at beginning of school year; (2) school psychologist to assess students identified as 'at-risk'  School-wide screening: (1) self-report rating scale; (2) school psychologist to assess students identified as 'at-risk'  Results fed back to parents (for at-risk students only) with referral information | School psychologists: n = 211 (31.7% male) | NA |
| **Miller (1999); USA**  **Suicide risk** | Cross- sectional | To explore high school principals' perceptions of three different models of school-based suicide prevention programmes. | High school  ND | NA | Curriculum based: (1) school psychologist to provide information on suicide (warning signs, incidence, etc.) in 2-hr slot; (2) school psychologist to assess students identified as 'at-risk'  Staff in-service training: (1) staff receive 2-hr presentation on suicide prevention at beginning of school year; (2) school psychologist to assess students identified as 'at-risk'  School-wide screening: (1) self-report rating scale; (2) school psychologist to assess students who scored above predetermined cut-off  Results fed back to parents (for at-risk students only) with referral information | High school principals: n = 185 (82.8% male) | NA |
| **Scherff (2005); USA**  **Suicide risk** | Cross- sectional | To explore school superintendents’ perceptions of three different models of school-based suicide prevention programmes. | High school  ND | NA | Curriculum based: (1) school psychologist to provide information on suicide (warning signs, incidence, etc.) in 2-hr slot; (2) school psychologist to assess students identified as 'at-risk'  Staff in-service training: (1) staff receive 2-hr presentation on suicide prevention at beginning of school year; (2) school psychologist to assess students identified as 'at-risk'  School-wide screening: (1) self-report rating scale; (2) school psychologist to assess students who scored above predetermined cut-off  Results fed back to parents (for at-risk students only) with referral information | School superintendents: n = 210 (79.4% male) | NA |
| **Whitney (2011); USA**  **Suicide risk** | Qualitative | To explore school principals' perceptions of school-wide identification models by examining three different models.   To examine barriers of implementation. | Elementary school Middle school High school  ND | NA | Curriculum-based: (1) school psychologist to provide information on suicide (warning signs, incidence, etc.) in ~2-hr slot; (2) school psychologist/counsellor to assess students identified as 'at-risk'  Staff in-service training: (1) **all** staff receive ~2-hr training on suicide prevention at beginning of school year from school psychologist/ counsellor; (2) school psychologist to assess students identified as 'at-risk'  School-wide screening: (1) brief (~10 min) self-report rating scale; (2) school psychologist/ counsellor to assess students who scored above predetermined cut-off  Results fed back to parents (for at-risk students only) with referral information | Public school principals: n = 7 (5/7 males; 3 high school, 1 middle school, 2 elementary school, 1 K-2 primary school) | NA |

NA = not applicable; ND = not described

^1^ Study designs represent the designs used to measure feasibility.
